# Marine-Derived Macrolides 1990–2020: An Overview of Chemical and Biological Diversity

**DOI:** 10.3390/md19040180

**Published:** 2021-03-25

**Authors:** Hairong Zhang, Jiabin Zou, Xiaoxue Yan, Junlong Chen, Xiujiao Cao, Jialing Wu, Yinghui Liu, Tingting Wang

**Affiliations:** Li Dak Sum Marine Biopharmaceutical Research Center, Department of Marine Pharmacy, College of Food and Pharmaceutical Sciences, Ningbo University, Ningbo 315800, China; heazin@163.com (H.Z.); 15370044785@163.com (J.Z.); yxxdyouxiang@163.com (X.Y.); chenjunlong2320@yahoo.com (J.C.); caoxiujiaoxx@163.com (X.C.); wujialing2020@126.com (J.W.); alltimed@163.com (Y.L.)

**Keywords:** macrolides, marine organisms, chemical diversity, biological diversity, cytotoxicity

## Abstract

Macrolides are a significant family of natural products with diverse structures and bioactivities. Considerable effort has been made in recent decades to isolate additional macrolides and characterize their chemical and bioactive properties. The majority of macrolides are obtained from marine organisms, including sponges, marine microorganisms and zooplankton, cnidarians, mollusks, red algae, bryozoans, and tunicates. Sponges, fungi and dinoflagellates are the main producers of macrolides. Marine macrolides possess a wide range of bioactive properties including cytotoxic, antibacterial, antifungal, antimitotic, antiviral, and other activities. Cytotoxicity is their most significant property, highlighting that marine macrolides still encompass many potential antitumor drug leads. This extensive review details the chemical and biological diversity of 505 macrolides derived from marine organisms which have been reported from 1990 to 2020.

## 1. Introduction

The term “macrolide” was coined by Woodward in 1957 [1] to describe antibiotics which typically consist of 14-, 15- or 16-membered macrolactam rings and feature double bonds and different saccharide and aminosaccharide functional groups. The naturally occurring 14-membered lactones erythromycin and clarithromycin, 15-membered macrolides azithromycin and spiramycin, and the 16-membered avermectin B1a are typical macrolide antibiotics in clinical use [2,3,4]. The 26-membered macrolide oligomycin A (an inhibitor of ATP synthase) [5,6] and the 36-membered macrocyclic lactone amphotericin B (an antifungal agent) are also used clinically [7,8]. In the last thirty years, many studies have described the molecular features, structures, and bioactivities of the intriguing macrolides obtained from plants, animals, and microbes in terrestrial and marine ecosystems [9,10,11,12]. Macrolides with larger macrocyclic rings have been reported, exemplified by the cytotoxic swinholide H, with its 40-membered lactone ring, obtained from the New Zealand deep-water marine sponge *Lamellomorpha strongylata* (*La. strongylata*) [13], and the novel 62-membered polyol symbiodinolide from the symbiotic dinoflagellate *Symbiodinium* sp. [14]. Macrolides, therefore, can be considered more broadly as a class of uncorrelated compounds containing a ring of twelve or more members.

**Figure 1:**
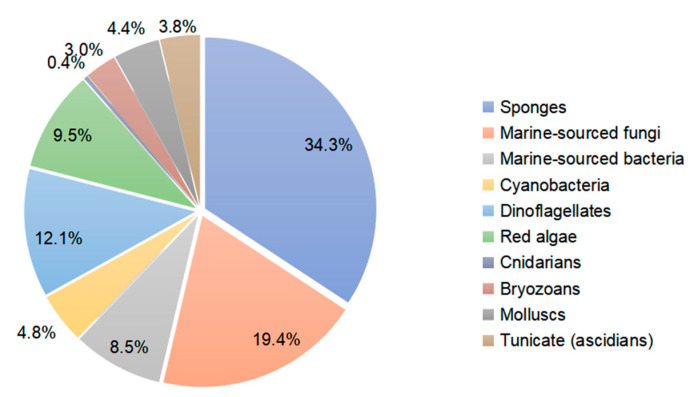
The percentage of macrolides from diverse marine organisms.

**Figure 2:**
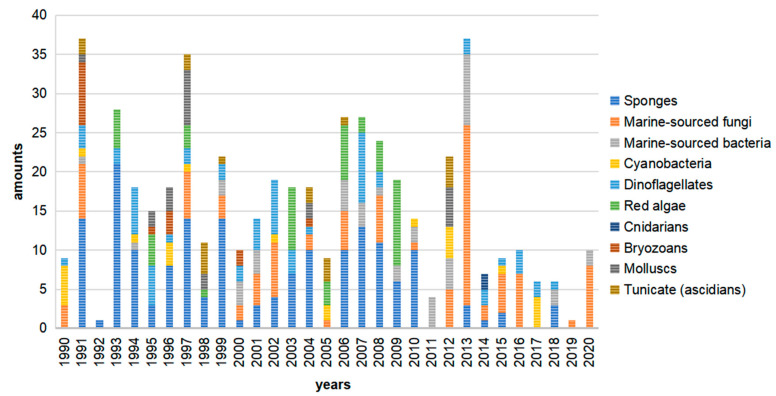
All new macrolides by source/year, *n* = 505.

This literature review from 1990 to 2020 highlights 505 new macrolides derived from marine organisms (65.8% of which are from sponges, fungi, and dinoflagellates) (Figure 1). Compared with terrestrial environments, the oceans exhibit more wide-ranging hypersaline, hyperbaric, hypoxic, cryogenic, and oligotrophic conditions. Marine organisms must develop the capacity to produce diverse bioactive metabolites to survive in these complex and competitive ecosystems. Marine metabolites have huge potential as new drug leads, with nine approved pharmaceuticals and 31 compounds in clinical pharmaceutical trials [15]. Macrolides are a significant family of natural marine products (Figure 2). The marine macrolides reviewed herein display cytotoxic, antibacterial, antifungal, antimitotic, antiviral, antiplasmodial and other bioactivities, as listed in Table 1. This review discusses the isolation, structures, and chemical and bioactive diversity of marine macrolides from 309 publications. 

## 2. Chemical and Biological Diversity of Marine-Derived Macrolides 

### 2.1. Macrolides Extracted from Marine Organisms

#### 2.1.1. Sponges

The Okinawan *Theonella* sp. (*T.* sp.) sponges produce**d** a series of dimeric macrolides called swinholides A–G (**1**–**7**) and isoswinholide A (**8**) [16,17,18,19]. Four bistheonellide-related compounds—bistheonellide C (**9**), isobistheonellide A (**10**), and bistheonellic acids A (**11**) and B (**12**)—are also produced by Okinawan *T.* sp. sponges [20]. The structure of the macrolide miyakolide (**13**), which is weakly cytotoxic and obtained from Japanese sponge *Polyfibrospongia* sp., was elucidated by X-ray single crystal diffraction [21]. 13-Deoxytedanolide (**14**) was isolated from *Mycale adhaerens* (*M. adhaerens*) and identified by spectroscopic analysis [22]. 


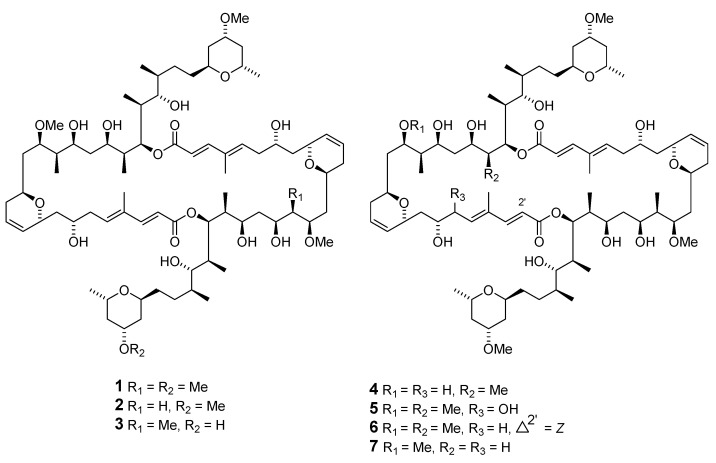



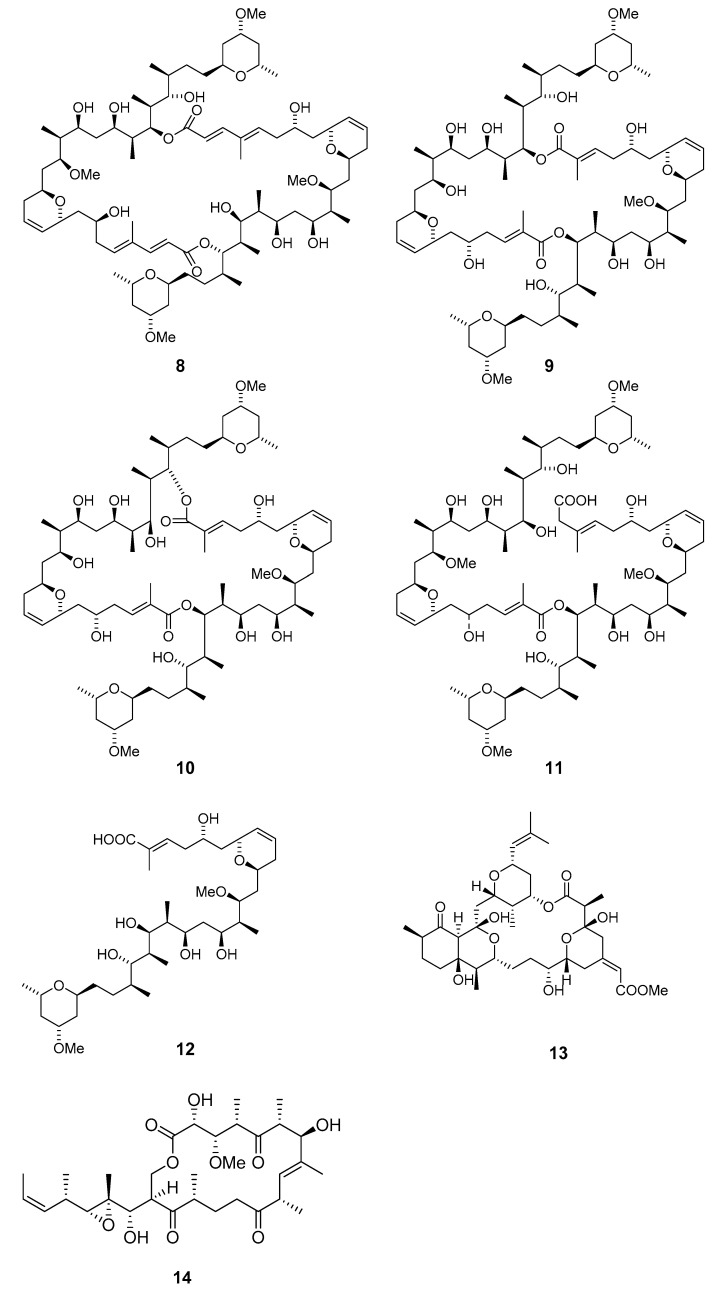


The antimitotic macrolides halistatin 1 (**15**) and halistatin 2 (**16**) were isolated from *Phakellia carteri* from the Comoros Islands and *Axinella* cf. *carteri* (Dendy) from the Western Indian Ocean [23,24]. Halistatin 3 (**17**) was produced in extremely small quantities by *Phakellia* sponges collected at Chuuk [25]. 


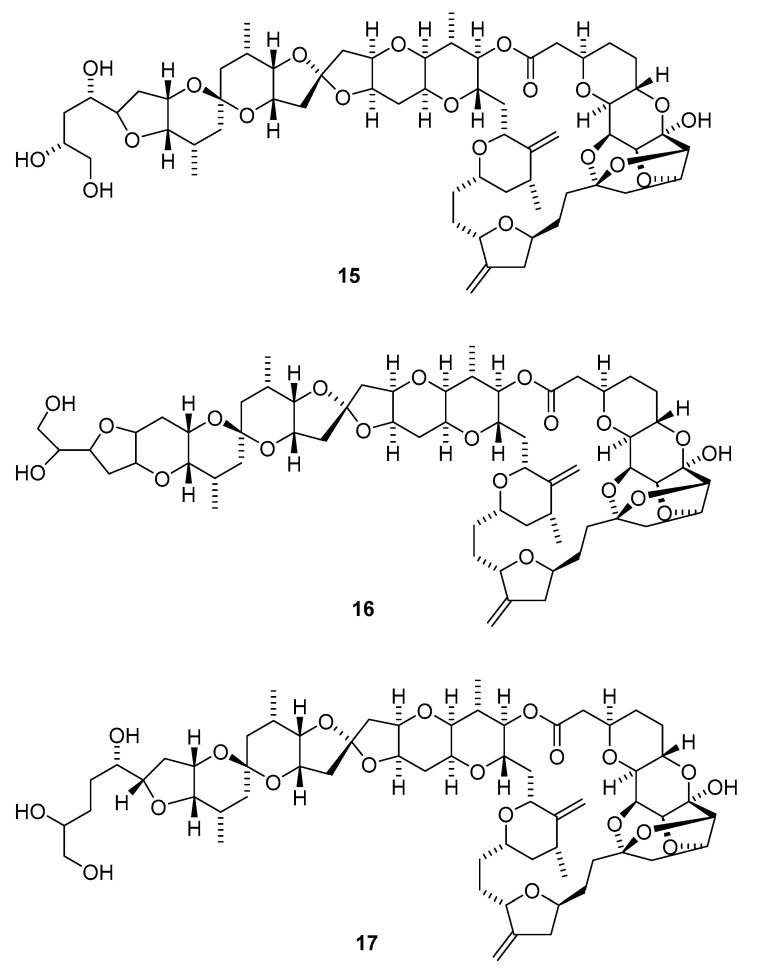


Independent groups have reported the potent antitumor macrolides spongiastatins 1 (**18**), 2 (**19**), and 3 (**20**), which were isolated from *Spongia* sp. in the Republic of Maldives and identified via spectral data without stereochemistry [26,27]. Another group isolated spongiastatin congeners 4 (**21**), 5 (**22**), 6 (**23**), 7 (**24**), 8 (**25**), and 9 (**26**) from *Spirastrella spinispirulfera* (*S. spinispirulfera*) on the southeast coast of Africa [28,29].


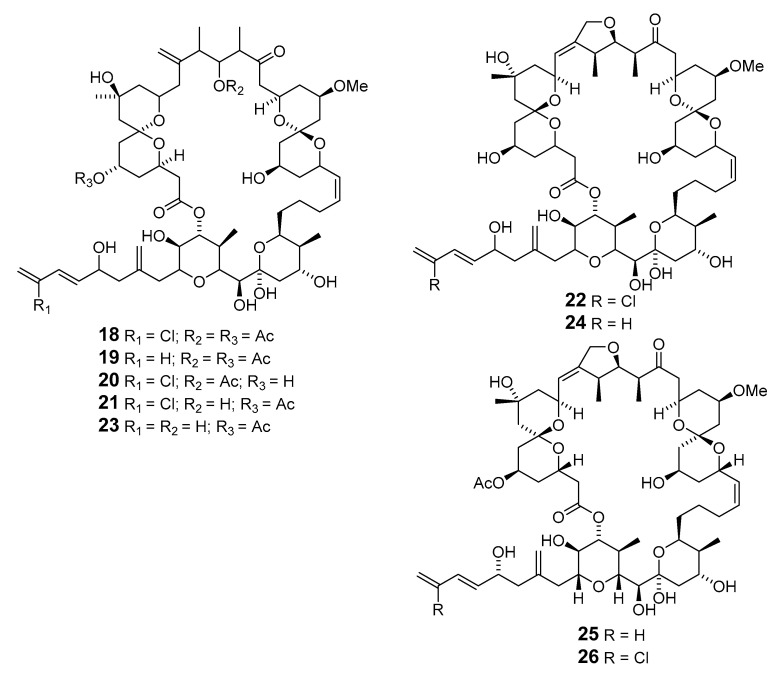


Three macrolides sphinxolides B (**27**), C (**28**), and D (**29**) have been isolated from the Caledonian sponge *Neosiphoniu superstes* [30]. 


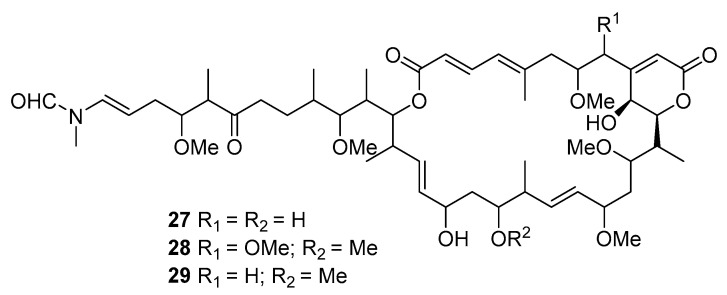


Three new trisoxazole macrolides, jaspisamides A (**30**), B (**31**), and C (**32**), were reported without stereochemical data in an Okinawan *Juspis* sponge [31]. 


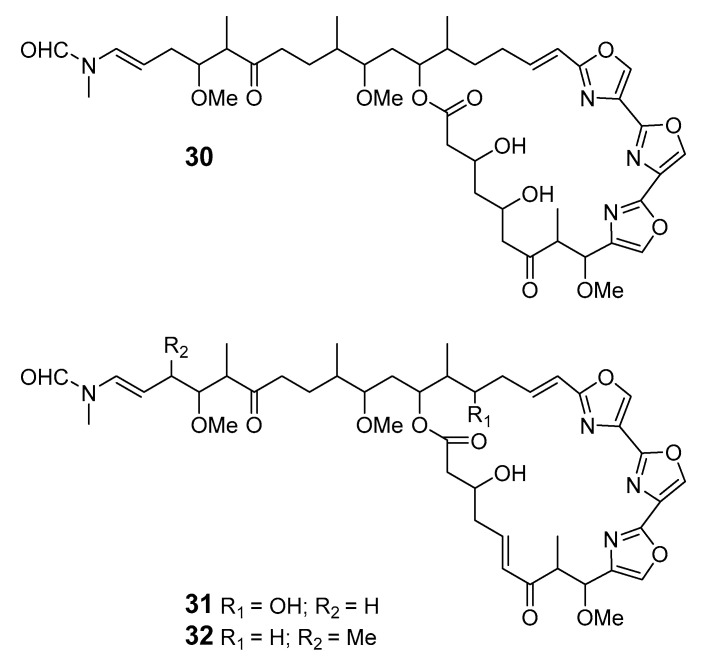


A new 22-membered macrocyclic lactone named dictyostatin 1 (**33**) was isolated from a Republic of Maldives *Spongia* sponge and exhibited significant cytotoxicity towards murine P388 lymphocytic leukemia [32]. The relative stereochemistry of dictyostatin 1 was determined by Murata’s method [33]. Two new 26-membered macrolides, reidispongiolides A (**34**) and B (**35**), have been produced by the marine sponge *Reidispongia coerulea* (*R. coerulea*) [34]. The relative and absolute stereochemistries of the C-23–C-35 portion of reidispongiolide A were determined by synthesis of an ozonolysis fragment of the natural product [35], which was later synthesized enantioselectively [36]. The relative stereochemistry of the C-7–C-15 fragment was reassigned through a series of diastereomers of a degradation fragment synthesis [37]. 


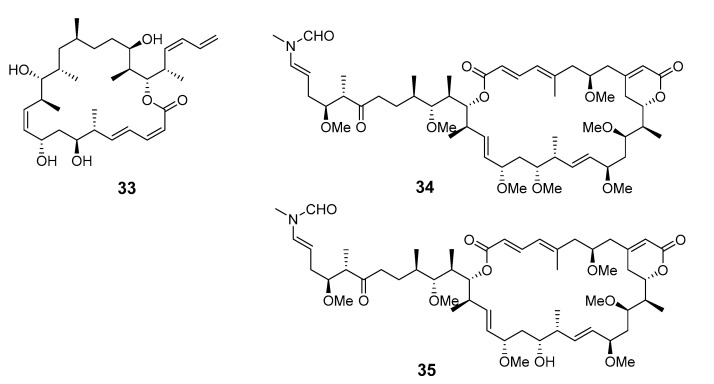


Cytotoxic superstolide A (**36**) and superstolide B (**37**) have been isolated from the deep-water marine sponge *Neosiphonia superstes* (*N. superstes*) [38,39]. Another cytotoxic macrolide, lasonolide A (**38**), was produced by the shallow-water Caribbean sponge *Forcepia* sp. [40]. Isohomohalichondrin B (**39**), belonging to the halichondrin family, was isolated from the New Zealand deep-water sponge *Lissodendoryx* sp. (*Li.* sp.) [41]. Phorboxazoles A (**40**) and B (**41**) have an unprecedented scaffold and were isolated from the Indian Ocean sponge *Phorbas* sp. (*P.* sp.), with complete stereochemistry and absolute configuration determined by spectroscopy and partial synthesis [42,43]. The structures and absolute configurations of latrunculin A (**42**) and laulimalide B (**43**) isolated from Okinawan sponge *Fasciospongia rimosa* were determined by X-ray analysis [44]. Other cytotoxic macrolides, latrunculin S (**44**), neolaulimalide (**45**) and zampanolide (**46**), have been produced by the *F. rimosa* genus [45,46]. Halichlorine (**47**), isolated from the marine sponge *Halichodria okadai*, exhibited significant inhibition of vascular cell adhesion molecule 1 (VCAM-1) [47].


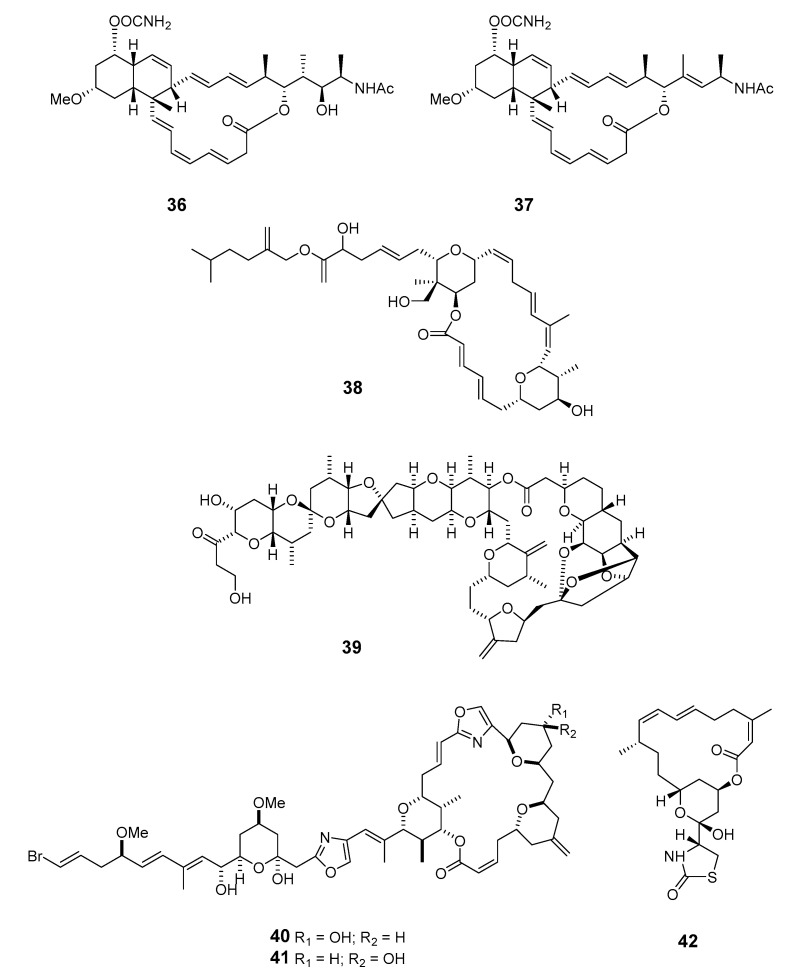



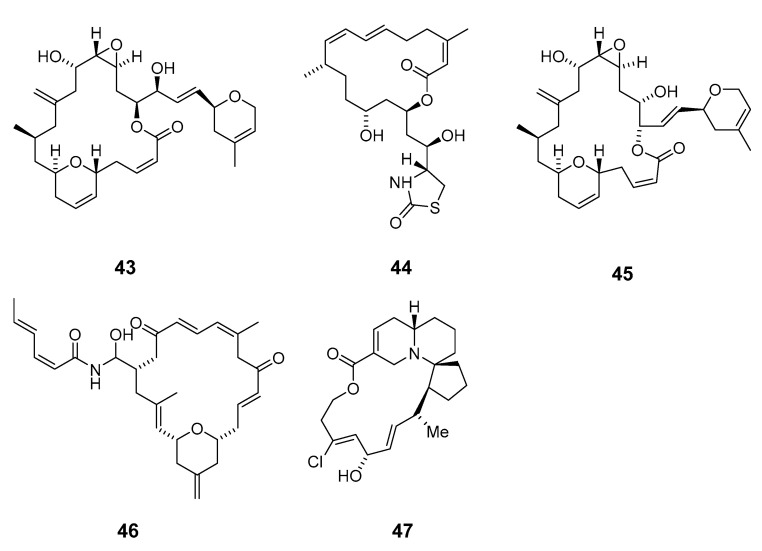


Leucascandrolide A (**48**), exhibiting antifungal and cytotoxic activities, was obtained from the sponge *Leucascandra caveolata* (*Le. caveolata*) [48]. The marine lithistida sponge *Callipelta* sp. (*Cal.* sp.) contains the first member of a new class of marine-derived macrolides, callipeltoside A (**49**), which incorporates an unusual chlorocyclopropyl group and an amino sugar [49]. The relative and absolute stereochemistry of the chlorocyclopropyl side chain of callipeltoside A was determined by stereoselective synthesis [50,51,52]. Cytotoxic macrolides altohyrtins A–C (**50**–**52**) and 5-desacetylaltohyrtin A (**53**) were isolated from the sponge *Hyrtios altum* and their absolute stereochemistries were determined by spectroscopy [53,54]. Screening of extracts from a New Zealand deep-water sponge *La. strongylata* for cytotoxicity towards the P388 cell line yielded swinholide H (**54**) [13].


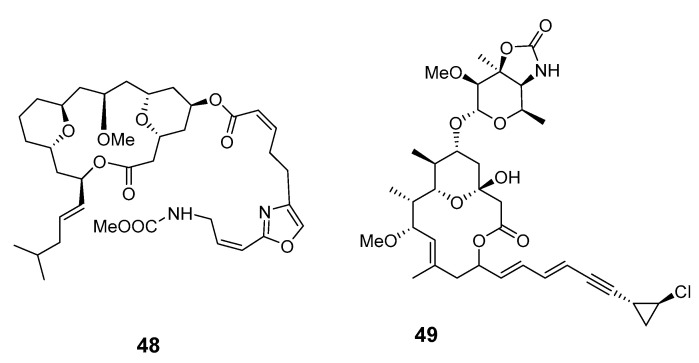



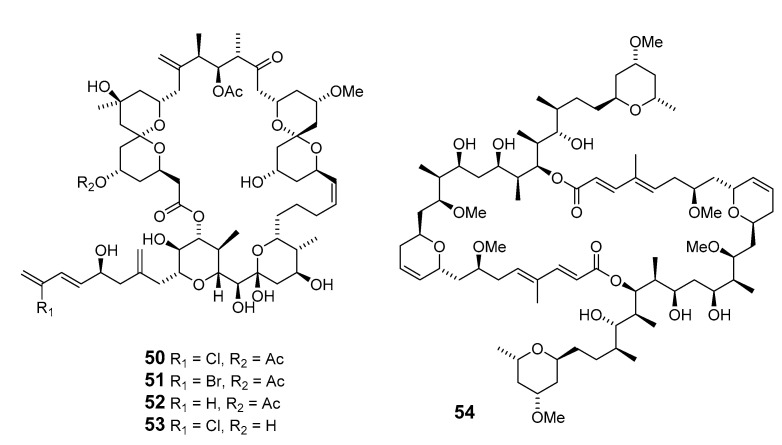


Another deep-water (> 100 m) sponge of the genus *Li.* produced the antitumor macrolides neonorhalichondrin B (**55**), neohomohalichondrin B (**56**), 55-methoxyisohomohalichondrin (**57**), 53-methoxyneoisohomohalichondrin B (**58a**) and 53-epi-53-methoxyneoisohomohalichondrin B (**58b**) [55]. 


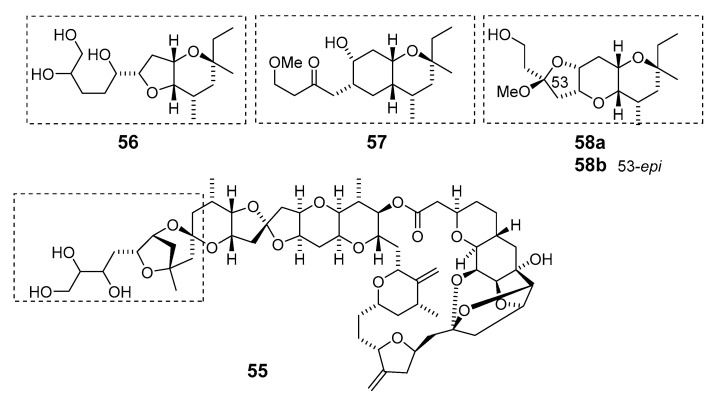


Macrolide salicylihalamides A (**59**) and B (**60**) were isolated from the *Haliclona* sponge, representing a potentially important new class of antitumor leads [56]. The absolute configurations of salicylihalamides A and B have been revised by a reinterpretation of Mosher ester derivatives and enantioselective syntheses of both enantiomers [57,58,59]. Cytotoxic callipeltoside B (**61**) and C (**62**), two members of a novel class of marine glycoside macrolides, were isolated from the sponge *Cal.* sp. [60].


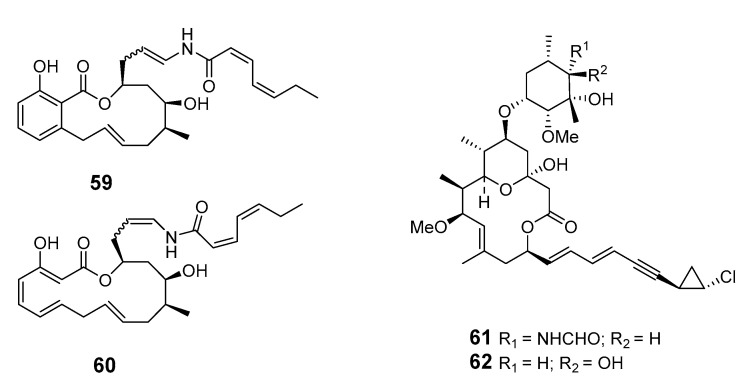


Four new oxazole-containing compounds, halishigamides A–D (**63**–**66**), were isolated from an Okinawan marine sponge, *Halichondria* sp. [61]. 


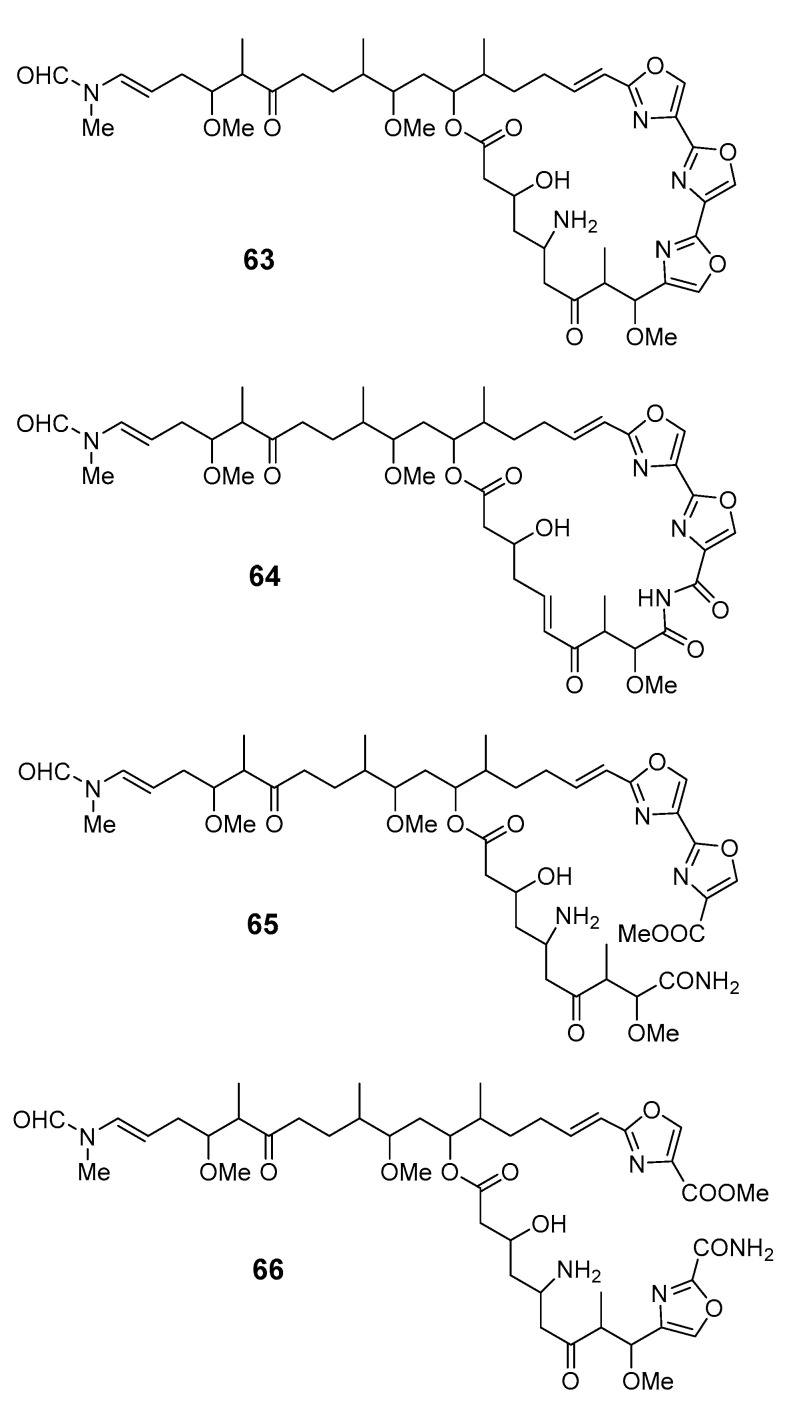


A Palau *Dysidea* sp. sponge contained a 14-membered macrolide, arenolide (**67**), showing modest cytotoxicity [62]. Three macrolides, 30-hydroxymycalolide A (**68**), 32-hydroxymycalolide A (**69**), and 38-hydroxymycalolide B (**70**), were isolated from the marine sponge *M. magellanica* and showed cytotoxicity towards L1210 cells [63]. 


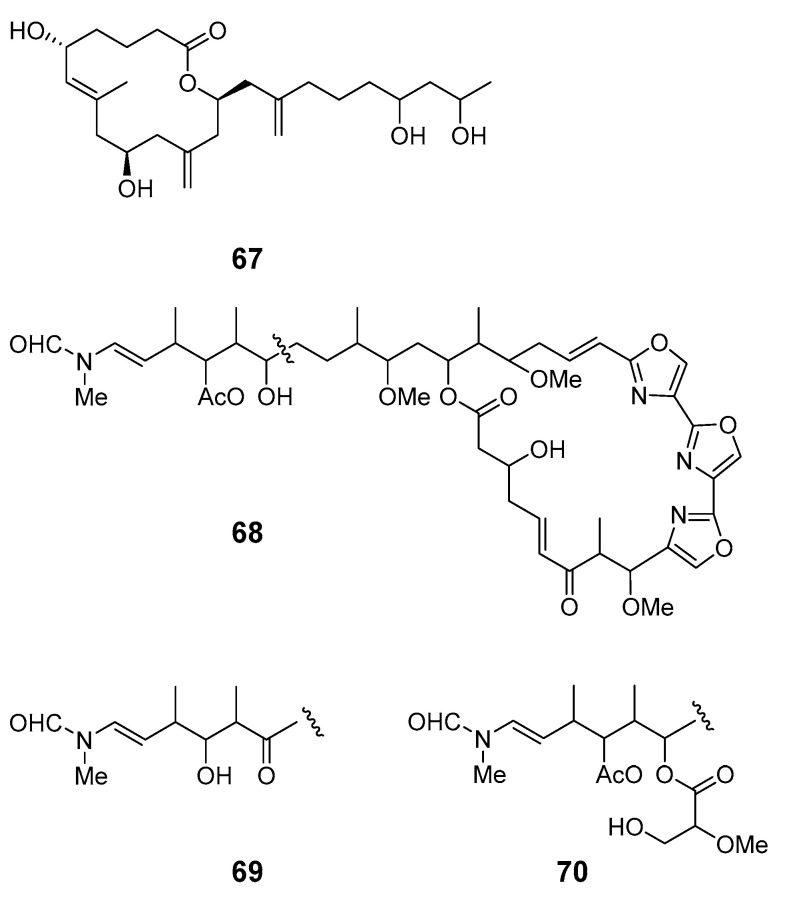


Pateamine 1 (**71**), a thiazole-containing macrolide with an unique dilactone functionality, was isolated from *M.* sp. sponge [64]. Four new macrocyclic lactones/lactams, amphilactams A–D (**72**–**75**), were produced by the marine sponge *Amphimedon* spp. collected in the Great Australian Bight [65]. 


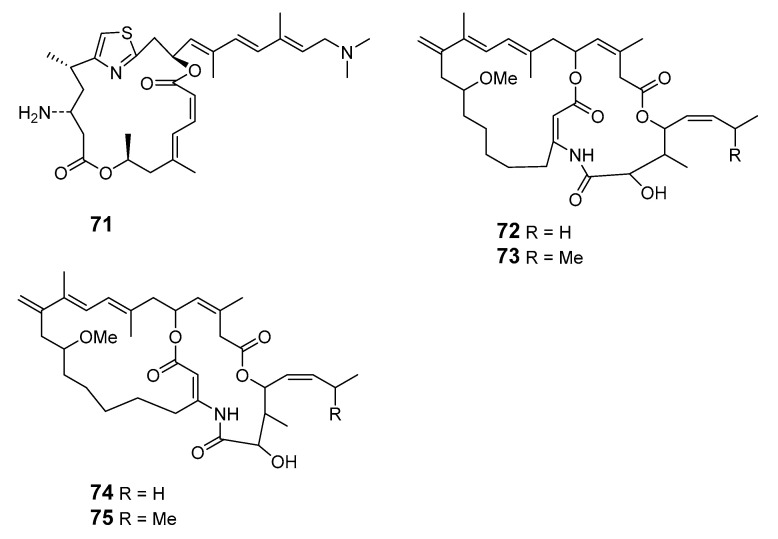


Cytotoxic macrolides haterumalides NA (**76**), NB (**77**), NC (**78**), ND (**79**) and NE (**80**) were isolated from the New Caledonian Litbistida sponges *N. superstes* and *R. Coerulea* [66]. Sphinxolides E–G (**81**–**83**) and reidispongiolide C (**84**) are new cytotoxic macrolides from Okinawan species of *Ircinia* [67]. Leucascandrolide B (**85**) is a 16-membered macrolide from the calcareous sponge *Le. caveolata* from the northeastern waters of New Caledonia [68]. The New Zealand marine sponge *M.* sp. contained the polyoxygenated, pyranose ring-containing, 16-membered macrolide peloruside A (**86**) [69], which was synthesized via a Mitsunobu-type lactonization [70]. 


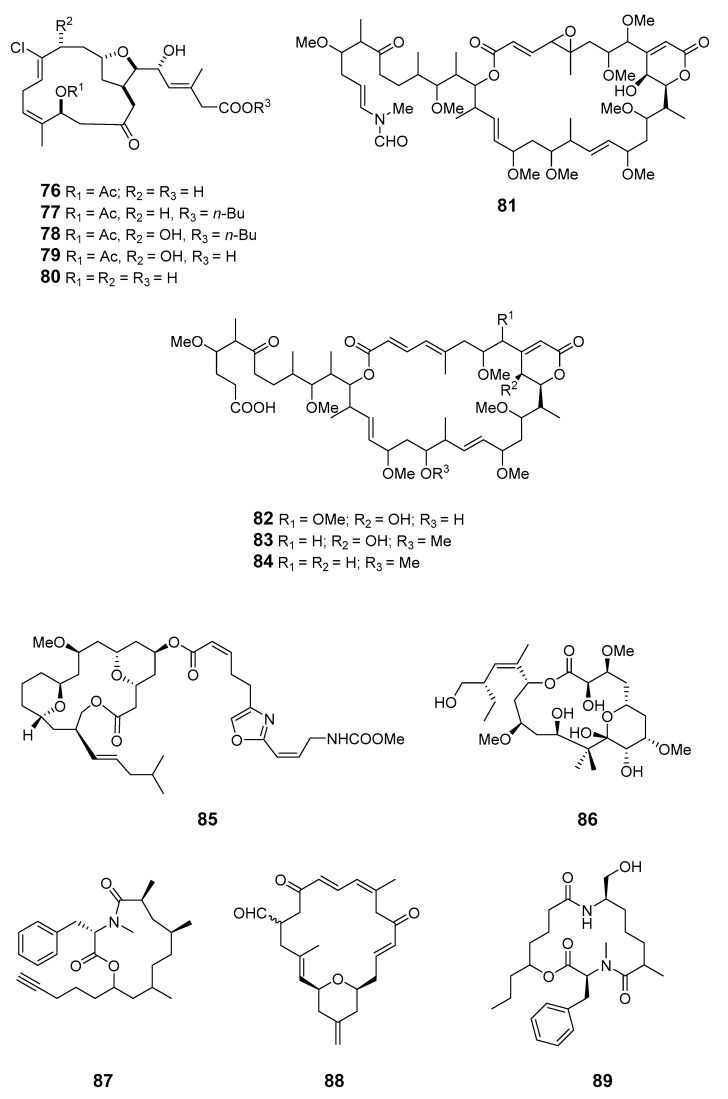


Cytotoxic spongidepsin (**87**) has been isolated from the Vanuatu marine sponge *Spongia* sp. [71]. A new cytotoxic 20-membered macrolide, dactylolide (**88**), was isolated from a marine sponge of the genus *Dactylospongia*. This has been synthesized and the relative stereochemistry of the acyloxymethine and the absolute configuration of the whole molecule have been determined [72]. The Vanuatu marine sponge *Ha.* sp. was found to contain the cyclic metabolite haliclamide (**89**) [73].

Clavosolides A–D (**90**–**93**) have been found in sponge *Myriastra clavosa* [74,75]. The absolute configurations of clavosides A and B were determined by total synthesis [74,75,76]. Spirastrellolides A–G (**94**–**100**) are antimitotic macrolides isolated from the Caribbean marine sponge *S. coccinea* [77,78,79,80]. Spirastrellolide A exhibited selective inhibition of protein phosphatase 2A [80].


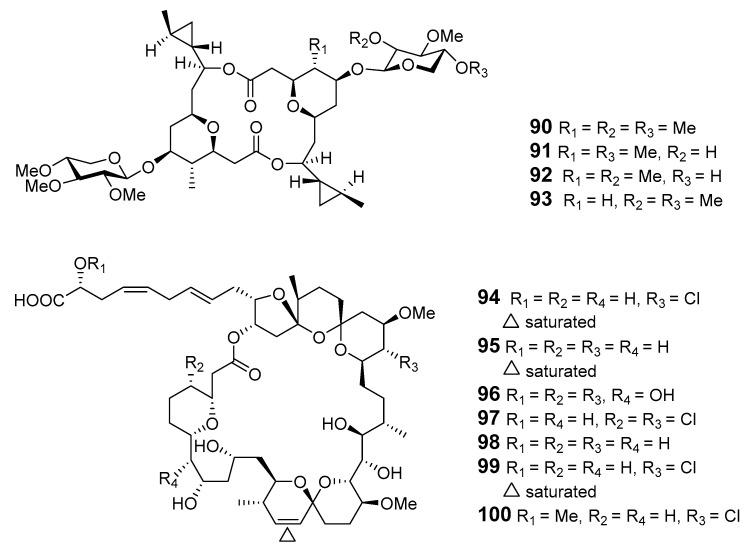


The sponge *Chondrosia corticata* produced two oxazole-containing macrolides, neohalichondramide (**101**) and (19*Z*)-halichondramide (**102**), and the open ringed secohalichondramide. Neohalichondramide and (19*Z*)-halichondramide exhibited significant cytotoxicity and antifungal activity toward the human leukemia cell-line K562 and Candida albicans (*C. albicans*) [81].


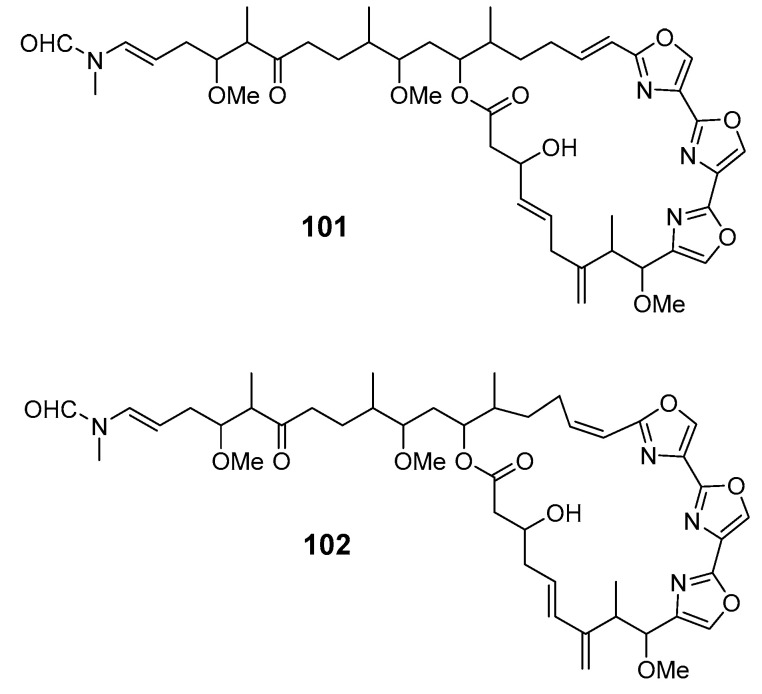


Three cytotoxic mycalolides, 30-hydroxymycalolide A (**103**), 32-hydroxymycalolide A (**104**) and 38-hydroxymycalolide B (**105**), have been isolated from a Japanese *M. magellanica* [82]. The five antiproliferative lasonolide congeners C–G (**106**–**110**) were isolated from *Forcepia* sponge collected in the U.S. Gulf of Mexico [83]. Exiguolide (**111**), isolated from the marine sponge *Geodia exigua*, was reported to inhibit fertilization of sea urchin (*Hemicentrotus pulcherrimus*) gametes but not embryogenesis [84]. The absolute configuration of exiguolide was determined by total synthesis of the enantiomer [85].


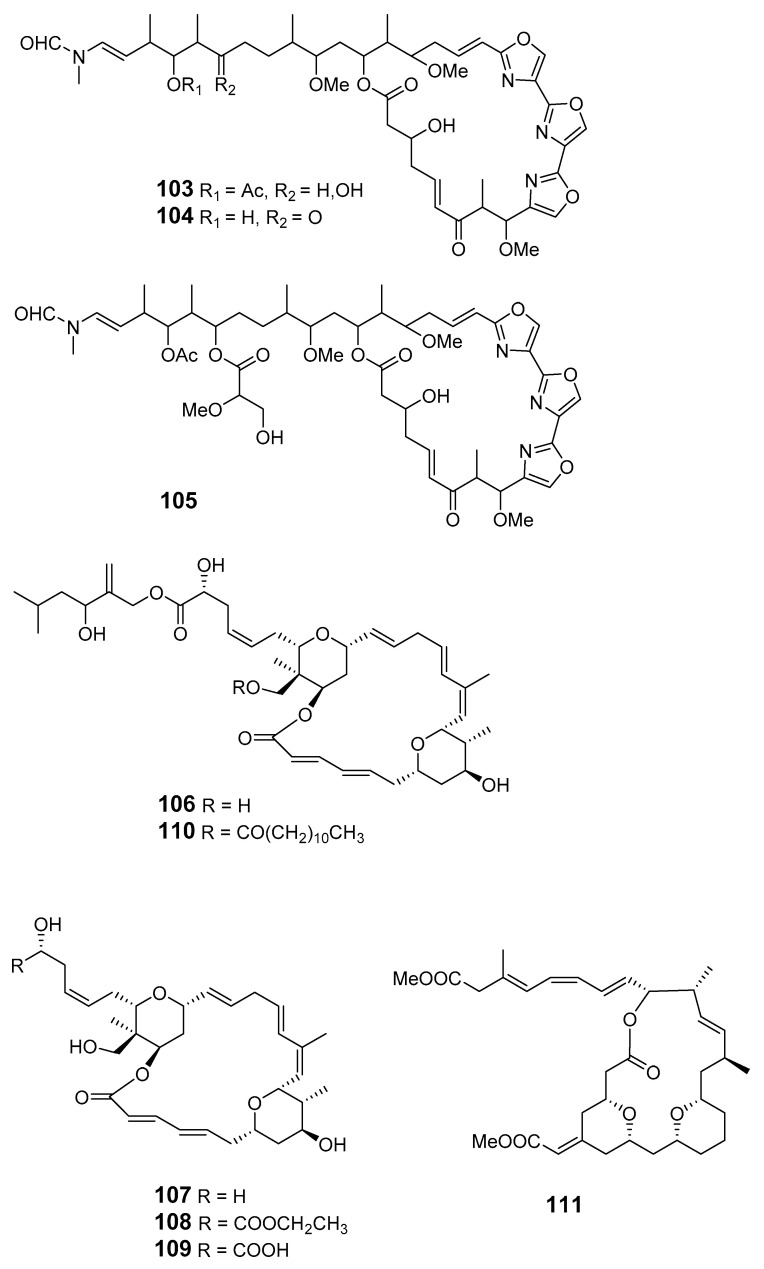


Cytotoxic macrolides leiodolides A (**112**) and B (**113**) were obtained from a deep-water (>200 m) *Leiodermatium* sponge [86,87]. Tedanolide C (**114**), isolated from *Ircinia* sp. (Papua New Guinea), was found to be potently cytotoxic, causing S-phase arrest, suggestive of protein synthesis inhibition [88]. Cytotoxic kabiramides F–I (**115**–**118**) were produced by *Pachastrissa nux* (*P. nux*) (Gulf of Thailand) [89]. 


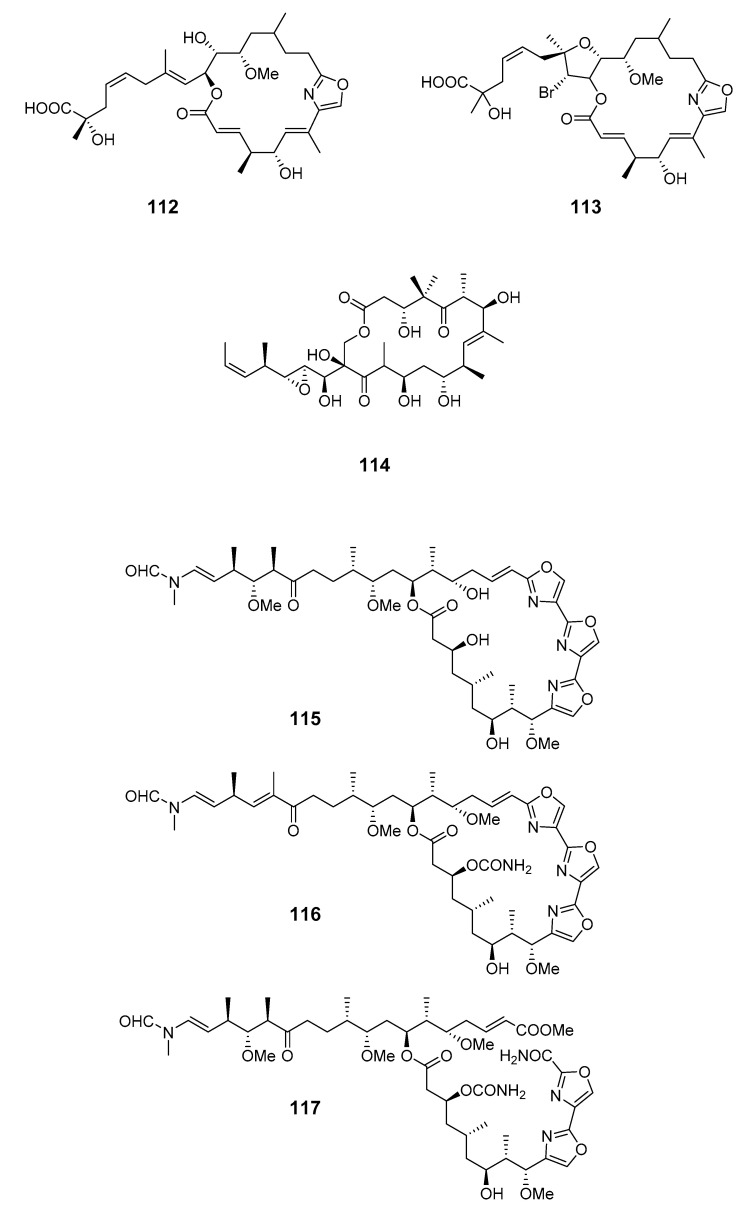



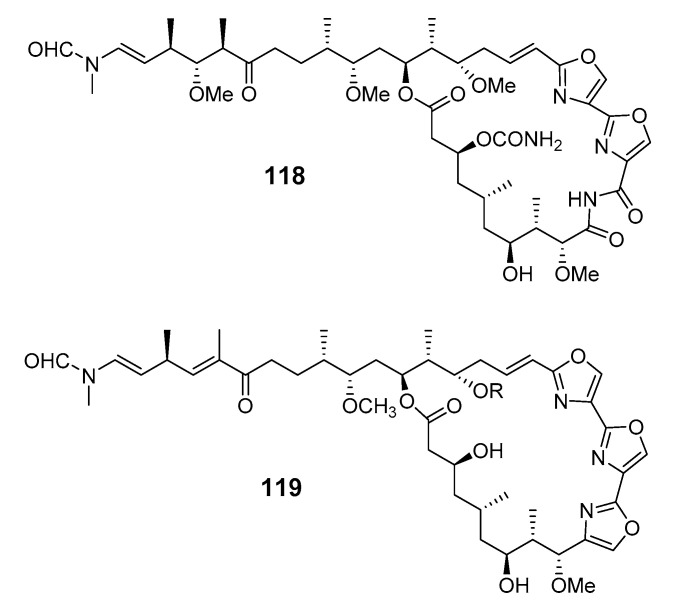


An antiplasmodial macrolide, kabiramide L (**119**), was isolated from *P. nux* sponge [90]. Swinholide I (**120**) and the related hurghadolide A (**121**), with cytotoxicity towards human colon cancer cells, were produced by *T. swinhoei* (Hurghada, Egypt) [91].


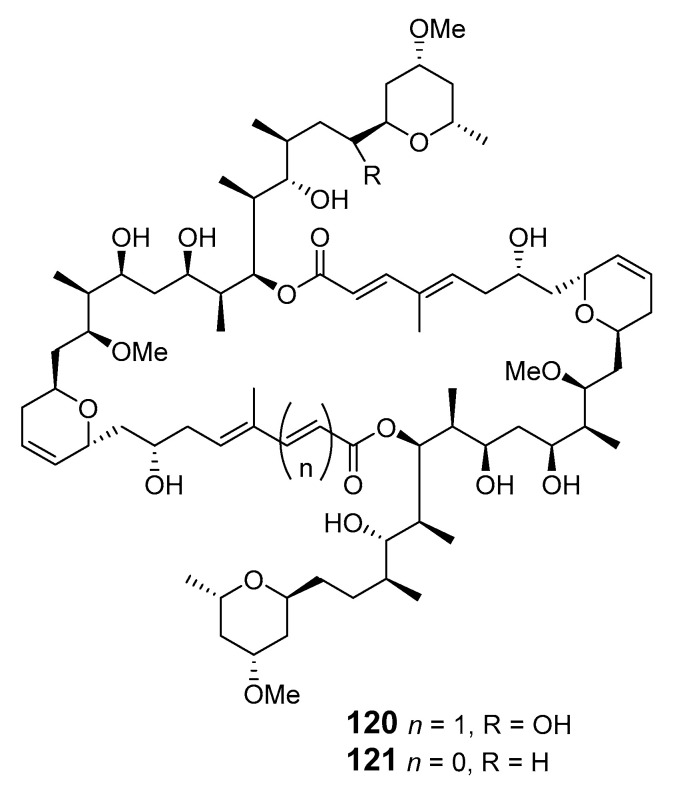


Oxalatrunculin B (**122**) was isolated from Red Sea sponge *Negombata corticata* and showed significant antifungal and anticancer activities, suggesting it as a potential member of the bioactive latrunculin family [92]. A Lithistid sponge of the family neopeltidae contained the macrolide neopeltolide (**123**) with potential cytotoxic and antifungal activities. This compound was synthesized to determine its absolute configuration and the relative stereochemistry of C-13 [93]. Candidaspongiolides (**124**), a complex mixture of acyl esters of a macrolide related to tedanolide, was isolated from *Candidaspongia* sp. (*Can.* sp.) (Papua New Guinea) and *Can. flabellata* (Great Barrier Reef, Australia) [94]. Fijianolides D–I (**125**–**130**) were produced by sponge *Cacospongia mycofijiensis* (*Cac. mycofijiensis*) (Mele Bay, Vanuatu) [95]. Phorbasides A–E (**131**–**135**) are chlorocyclopropane macrolides isolated from marine sponge *P.* sp. [96,97]. 


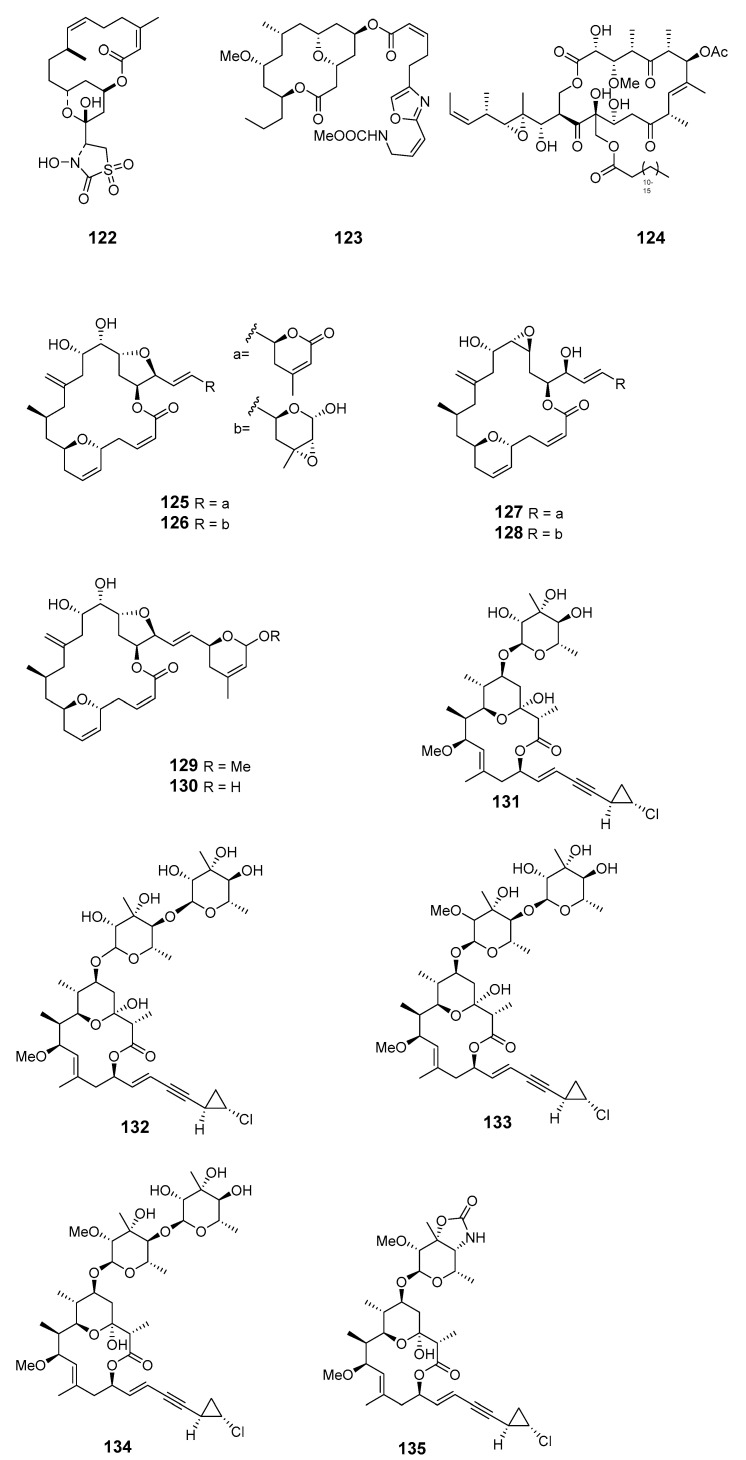


Latrunculin analogs, latrunculol A–C (**136**–**138**), 18-epi-latrunculol (**139**) and latrunculones A (**140**) and B (**141**), were obtained from *Cac. mycofijiensis* [98]. Salarin A (**142**), salarin B (**143**) and tulearin A (**144**) were obtained from repeated collections of the Madagascan sponge *Fascaplysinopsis* sp. (*F*. sp.) [99]. 


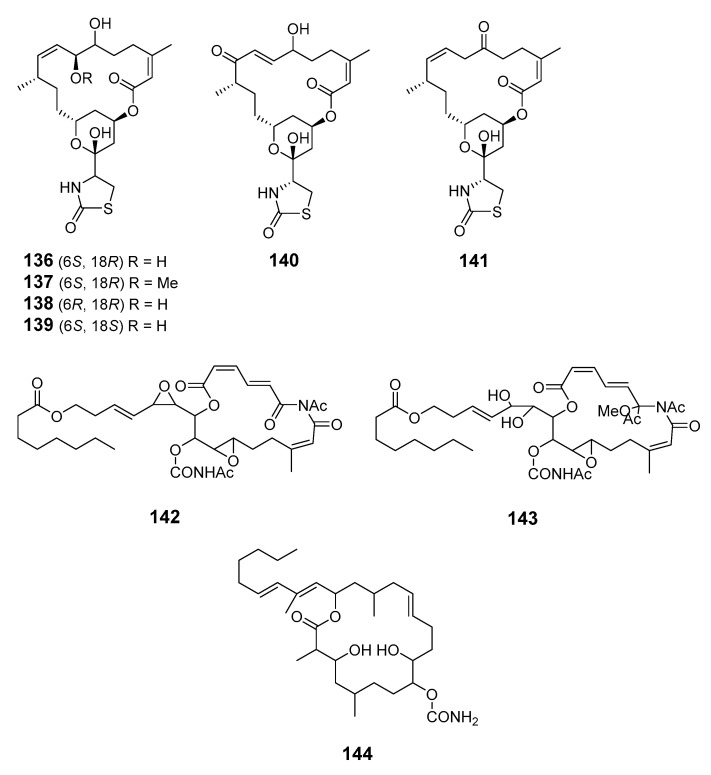


A further collection led to the isolation of salarin C (**145**), which was considered to be the precursor of salarins A and B [100]. Marine sponge *Siliquariaspongia mirabilis* contained an antitumor macrolide lactam named mirabilin (**146**) [101]. The nitrogenous bismacrolide tausalarin C (**147**) was isolated from the Madagascar sponge *F.* sp. and was found to inhibit proliferation of K562 leukemia cells [102]. Muironolide A (**148**), containing a rare hexahydro-1*H*-isoindolone and trichlorocarbinol ester, was isolated from marine sponge of the genus *Phorbas* [103]. 


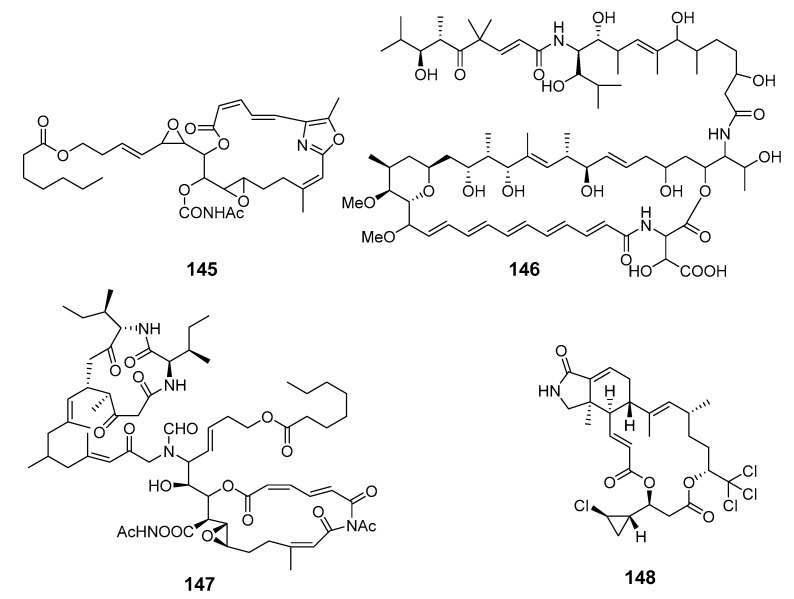


Four variants of halichondrin B, B-1140 (**149**), B-1092 (**150**), B-1020 (**151**) and B-1076 (**152**), were extracted from the Poecilosclerid sponge *Li.* sp. in microgram quantities and their structures were elucidated by capillary NMR spectroscopy [104]. 


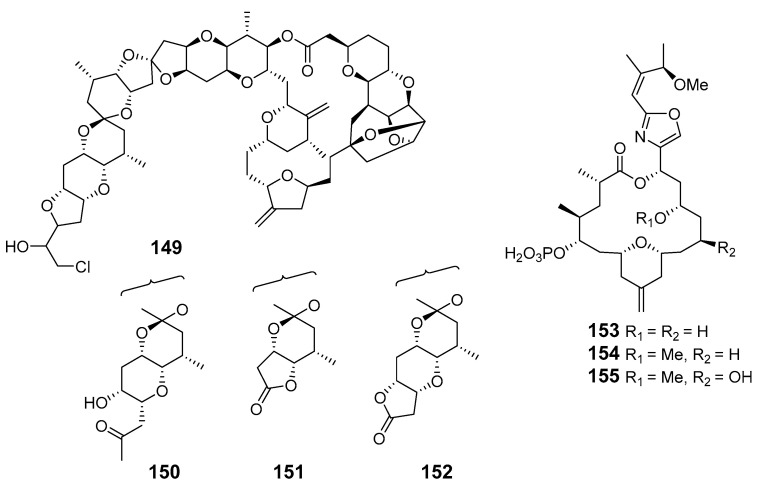


Cytotoxic phosphate-containing macrolide enigmazole A (**153**) and two analogs, 15-*O*-methylenigmazole A (**154**) and 13-hydroxy-15-*O*-methylenigmazole A (**155**), were extracted from the marine sponge *Cinachyrella enigmatica* collected in Papua New Guinea [105] and their structures were confirmed by total synthesis [106].

Seven scalarin analogs D–J (**156**–**162**) were obtained from the Madagascan *F.* sp. sponge. Scalarins D, E, H, and J inhibited cell proliferation in a dose- and time-dependent manner [107]. Theonezolides A–C (**163**–**165**) were obtained from the Okinawan marine sponge *T.* sp. and absolute configurations were determined by combining a JBCA method, a universal NMR database, and a ^13^C-acetonide method [108,109]. 


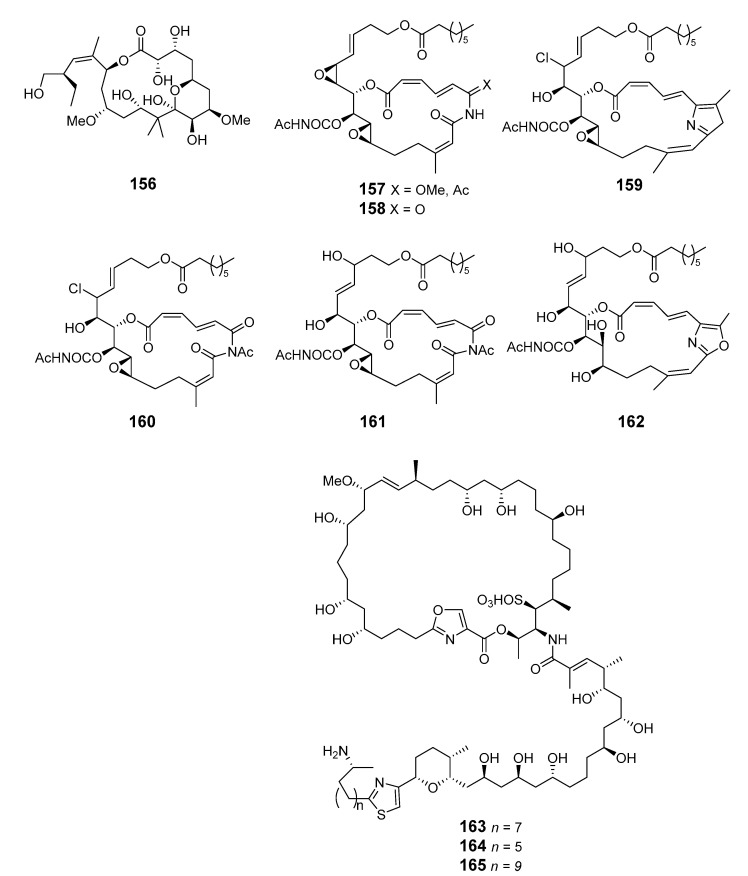


The Indonesian sponge *T. swinhoei* yielded the dimeric macrolides isoswinholide B (**166**) and swinholide K (**167**) [110]. An unusual carbamate, callyspongiolide (**168**), with strong cytotoxicity towards human Jurkat J16 T and Ramos B lymphocytes, was isolated from marine sponge *Cal.* sp. [111]. Cytotoxic polyketide macrolides phormidolides B (**169**) and C (**170**) were isolated from *Petrosiidae* sponge with stereochemical assignment via enantioselective synthesis of the macrocyclic core [112]. Cytotoxic chondropsin-type macrolides poecillastrins E (**171**), F (**172**), and G (**173**) were isolated from the marine sponge *Poecillastra* sp. [113].


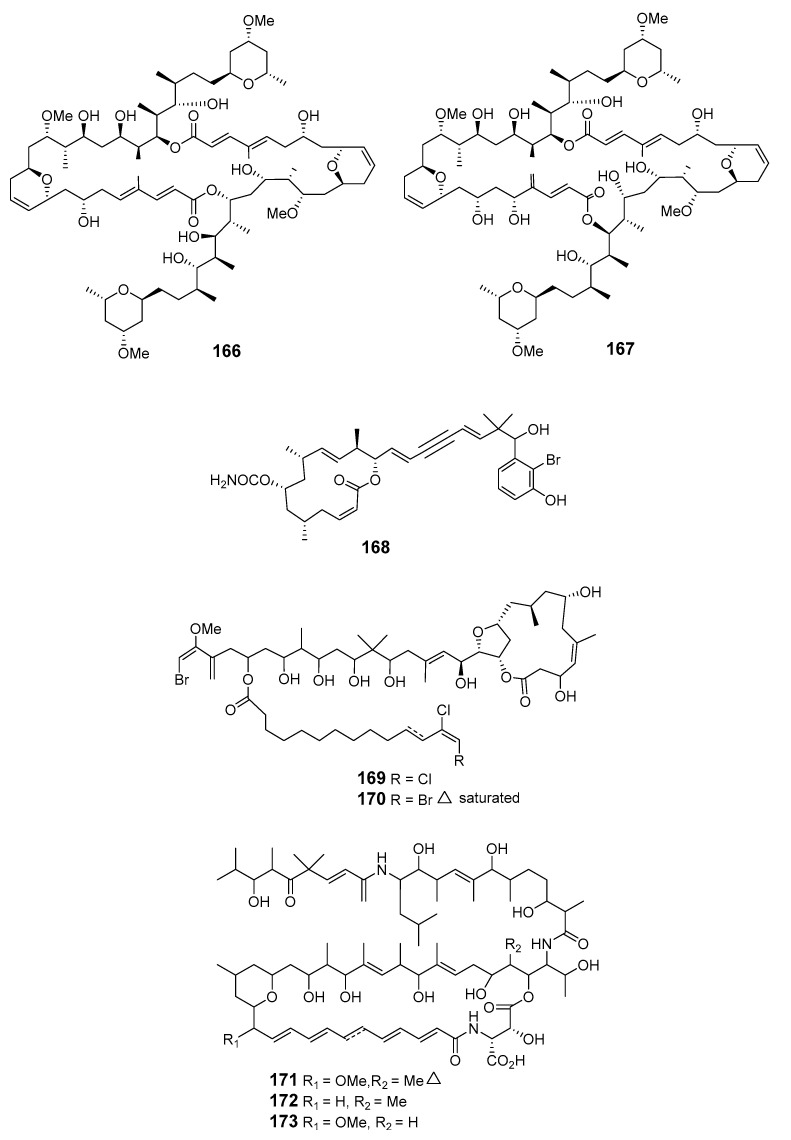


#### 2.1.2. Microorganisms and Zooplankton

##### Fungi

The fungus *Periconia byssoides* (*Per. byssoides*), obtained from the sea hare *Aplysia kurodai* (*Ap*. sp.), was reported to produce the cytotoxic triols pericosides A and B, and four new macrolides, macrosphelides E–H (**174**–**177**) [114]. Macrosphelide I (**178**) and macrosphelides E–H from *Per. byssoides* isolated from *Ap. kurodai* were also reported elsewhere [115]. Macrosphelide E was synthesized at a high yield via a key chemoenzymatic reaction [116]. The synthesis of macrosphelides H and G has also been described [117,118]. Absolute configurations determined by spectroscopy and chemical transformation have been reported for macrosphelides L (**179**) and H produced by *Per. byssoides* from *Ap. kurodai*, and the cytotoxic macrosphelide M (**180**) [114,119,120]. *Penicillum verruculosum* (IMI352119) was reported to produce three macrolides with antifungal activity: BK223-A (**181**), BK223-B (**182**) and BK223-C (**183**) [121]. The mitosporic fungus *Varicosporina ramulosa* has been reported to produce (6*R*,11*S*,12*S*,14*R*)-colletodiol (**184**), (6*R*,11*R*,12*R*,14*R*)-colletodiol (**185**) and colletoketol (**186**) [122,123]. The 12-membered macrolides pandangolide 1 (**187**) and pandangolide 2 (**188**) were extracted from an unidentified fungus isolated from marine sponge collected in Indonesia [124]. 


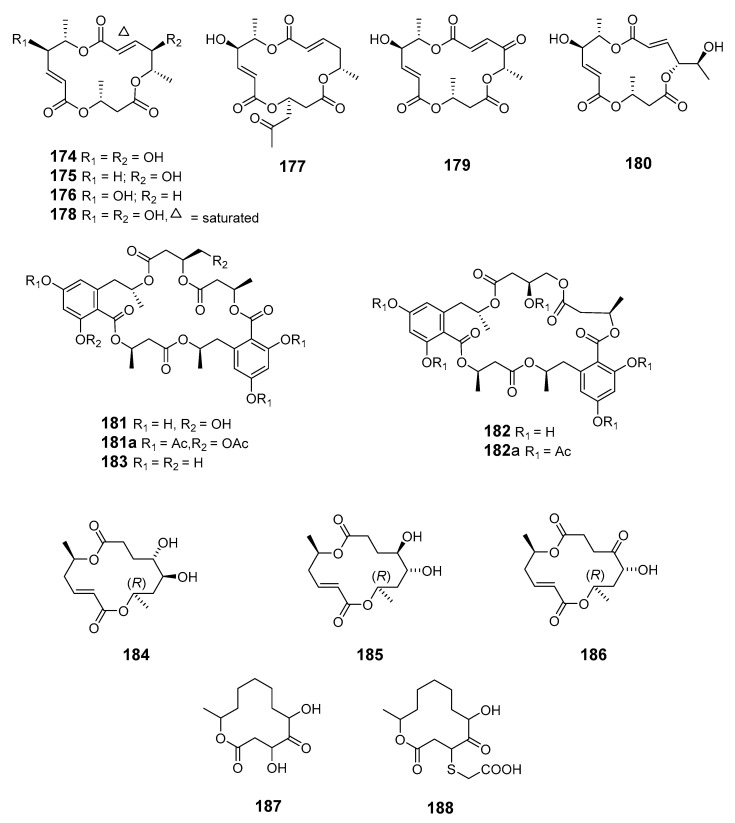


Pandangolide 3 (**189**), macrolide dimer pandangolide 4 (**190**), and a new acetyl derivative of 5-hydroxymethylfuran-2-carboxylic acid were produced by the fungus *Cladosporium herbarum* (*Cla. herbarum*), associated with the sponge *Callyspongia aerizusa* (*Cal. aerizusa*) and collected in Bali [125]. The cytotoxic macrocyclic trichothecene 12,13-deoxyroridin E (**191**) was obtained from an extract of the marine fungus *Myrothecium roridum* (*M. roridum*) [126]. The 14-membered resorcylic macrolides aigialomycins A–E (**192**–1**96**) were isolated from the mangrove fungus *Aigialus parvus* BCC 5311 [127]. Potential antifungal macrocyclic polyesters 15G256ɩ (**197**) and 15G256ѡ (**198**) were obtained from the marine fungus *Hypoxylon oceanicum* LL-15G256 [128]. Two cytotoxic macrolides, sporiolides A (**199**) and B (**200**), were produced by the fungus *Cladosporium* isolated from the brown alga *Actinotrichia fragilis* (Okinawa, Japan) [129]. 


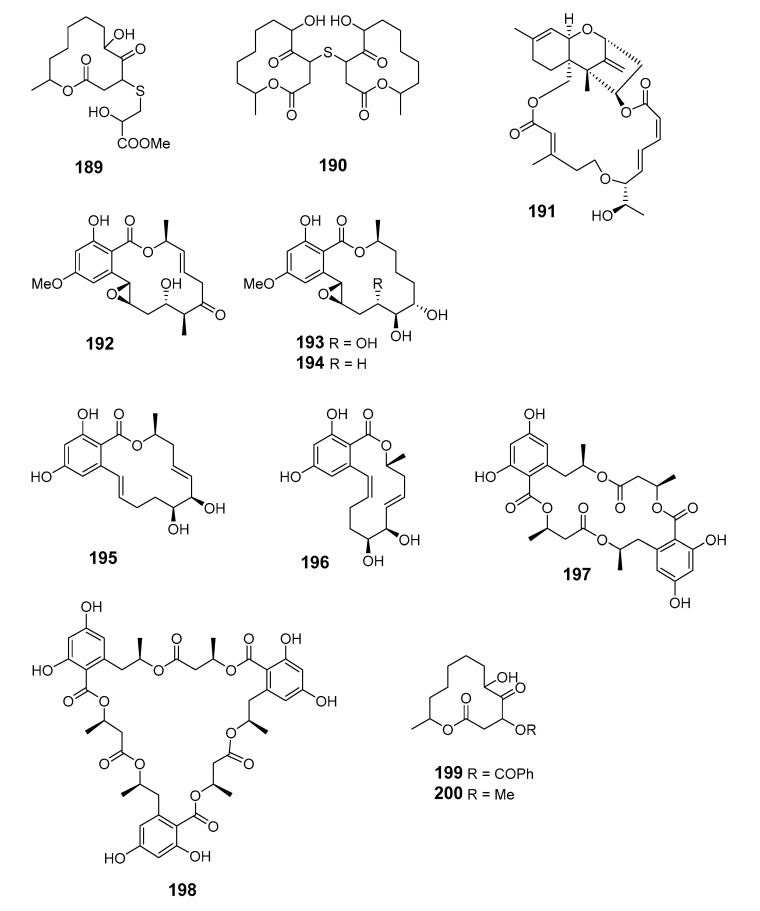


An unidentified endophytic fungus from the brown alga *Sargassum* sp. (Zhanjiang Sea, China) was the source of two 12-membered ring lactones (**201**–**202**) [130]. 12-Hydroxyroridin E (**203**), roridin Q (**204**) and 2,3-deoxyroritoxin D (**205**) were obtained from *M. roridum* on submerged wood in Palau [131]. *Gliocladium* sp. isolated from the alga *Durvillaea antarctica* (Tauranga Bay, New Zealand) yielded 4-ketoclonostachydiol (**206**) [132]. 


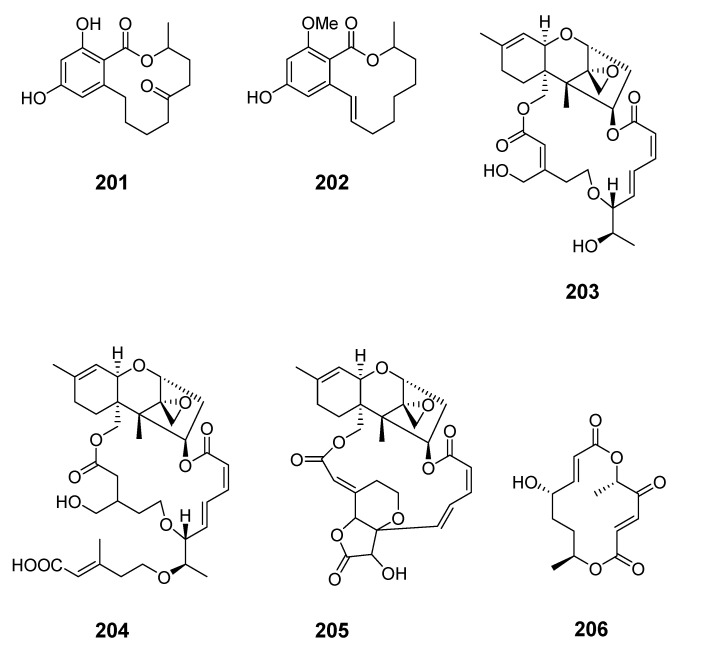


The 14-membered resorcylic acid lactone derivatives 8′-hydroxyzearalanone (**207**) and 2′-hydroxyzearalanol (**208**) were isolated from the marine-derived fungus *Penicillium* sp. (*Pen.* sp.) [133]. β-resorcylic macrolide 5′-hydroxyzearalenol (**209**) was obtained from the culture broth of the fungus *Fusarium* sp. 05ABR26 [134]. The cytotoxic 14-membered macrolides aspergillide A–C (**210**–**212**) were isolated from the culture broth of the marine sponge-derived fungus *Aspergillus ostianus* (*As. ostianus*) (Pohnpei, Micronesia) [135].


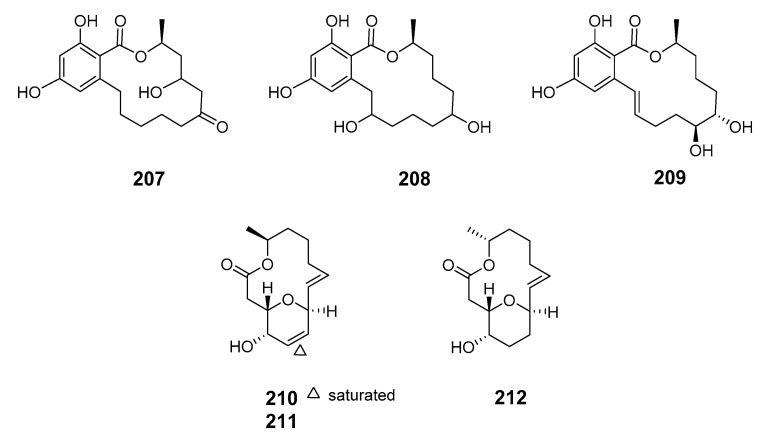


The marine-derived fungus *As.* sp. SCSGAF 0076 was reported to produce the 16-membered macrolide aspergillide D (**213**) [136]. Apralactone A (**214**) and enantiomers of curvularin (**215**–**220**) were isolated from *Curvularia* sp. (*Cur.* sp.) [137,138]. The macrolide curvulone A (**221**) was produced by *Cur.* sp. isolated from the marine alga *Gracilaria folifera* and inhibited the growth of *B. subtilis*, *Microbotryum violaceum*, *Septoria tritici*, and *Chlorella fusca* [139]. 


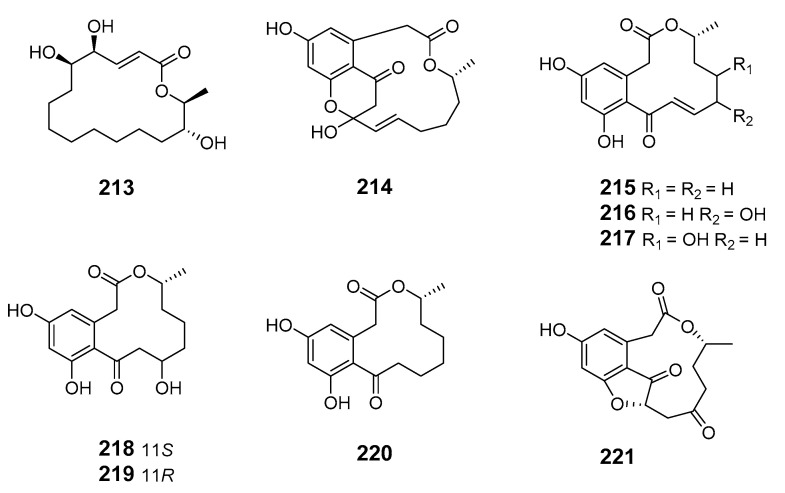


Three decalactones, xestodecalactones D–F (**222**–**224**), were purified from an ethyl acetate extract of *Corynespora cassiicola* isolated from leaf tissues of the Chinese mangrove medicinal plant *Laguncularia racemose* [140]. Seiricuprolide pestalotioprolides A (**225**) and B (**226**) (as the diacetate) were isolated from the fungus *Pestalotiopsis* spp., which is associated with mangrove twigs of *Rhizophora mucronata* [141]. Calcarides A–C (**227**–**229**), 15G256α (**230**), and 15G256β (**231**) were obtained from crude extracts of the fungus *Calcarisporium* sp. KF525 isolated from German Wadden Sea water samples [142]. 


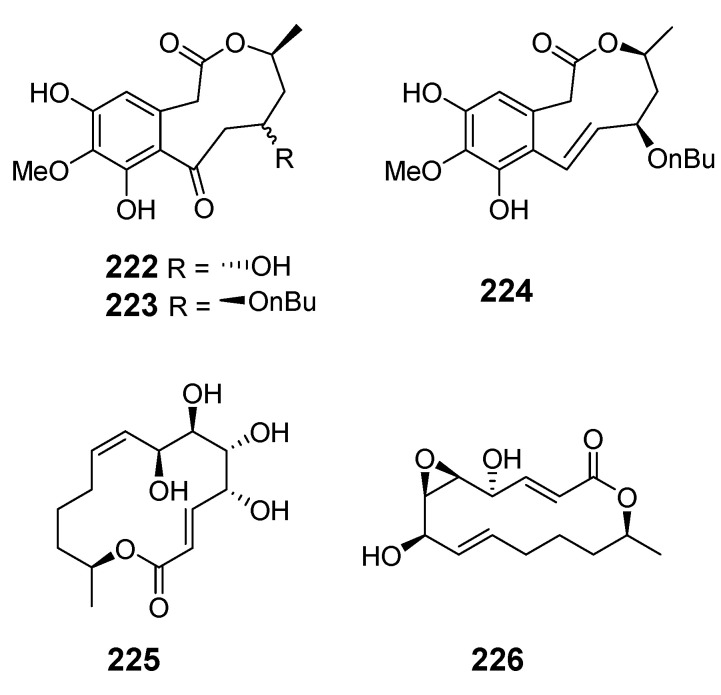



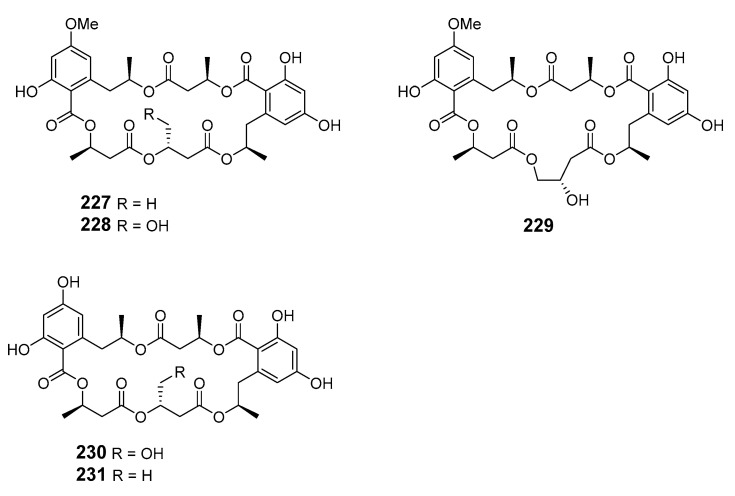


Thirteen new 12-membered macrolides, dendrodolides A–M (**232**–**244**), were obtained from the fungus *Dendrodochium* sp. derived from sea cucumber *Holothuria nobilis Selenka* in the South China Sea [143]. Dendrodolide K was obtained from a commercially available substrate by a convergent strategy, and the dendrolides F, G, I, J, and L were synthesized via a unified strategy employing ring-closing metathesis [144,145]. 


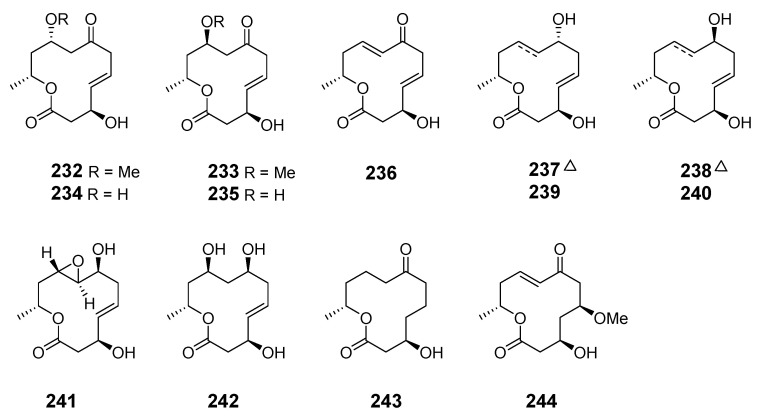


Cochliomycin C (**245**) was produced by the gorgonian-derived fungus *Cochliobolus lunatus* (*Coc. lunatus*) [146], its absolute configuration was corrected in a later study [147].


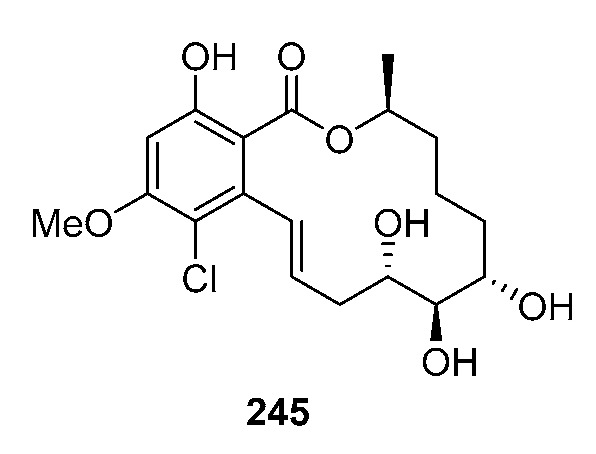


The fungus *Pen. sumatrense* MA-92, associated with the mangrove *Lumnitzera racemose*, yielded the sulfur-containing curvularin derivatives sumalarins A−C (**246**–**248**) [148]. Chemical epigenetic manipulation of the marine-derived fungus *Coc. lunatus* (TA26-46) with histone deacetylase inhibitors led to the elucidation of two 14-membered resorcylic acid lactones: 5-bromozeaenol (**249**) and 3,5-dibromozeaenol (**250**) [149]. Gliomasolides A–E (**251**–**255**) were obtained from a sponge-derived fungus *Gliomastix* sp. ZSDS1-F7-2, their structures being determined by spectroscopy and single crystal X-ray diffraction [150]. Two 13-membered macrolides (**256**–**257**) were isolated from the marine-derived fungus *Pen. meleagrinum* var. *viridiflavum* [151]. Application of published procedures for experimental design and chemometric analysis to enhance the production of curvularin-related compounds by marine-derived *Penicillium* sp. DRF2 led to the isolation of cyclothiocurvularins (**258**–**260**) and cyclosulfoxicurvularins (**261**–**262**) [152]. Thiocladospolide E (**263**) was produced by the mangrove endophytic fungus *Cladosporium* sp. (*Cla.* sp.) SCNU-F0001 and its absolute configuration was determined by X-ray diffraction [153]. Thiocladospolides F–J (**264**–**268**) were isolated from another mangrove-derived endophytic fungus species in the same *Cla.* genus [154]. The macrolide 6,7,9,10-tetrahydromutolide (**269**) was isolated from endophytic fungus *Aplosporella javeedii* [155]. Two trichothecene macrolides, myrothecines H and I (**270**–**271**), were obtained from the endophytic fungus *Paramyrothecium roridum* isolated from the medicinal plant *Morinda officinalis* [156].


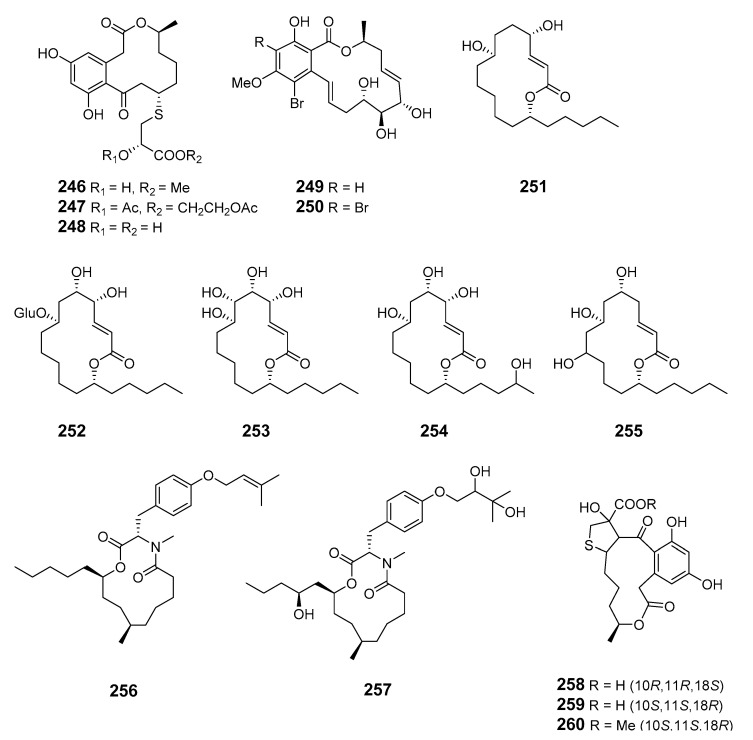



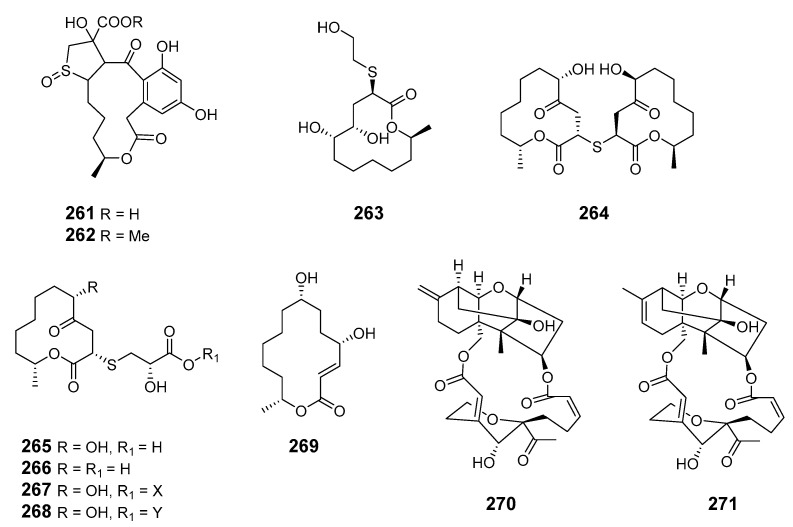


##### Bacteria

The 24-membered macrolide maduralide (**272**) was isolated from a marine bacterium in the order Actinomycetales [157]. Halichomycin (**273**) was produced by *Streptomyces hygroscopicus* (*S*. *hygroscopicus*) isolated from the marine fish *Halichoeres bleekeri* [158]. 7-*O*-Succinyl macrolactin F (**274**) and 7-*O*-succinyl macrolactin A (**275**) were isolated from a culture of marine *Bacillus* sp. (*B.* sp.) Sc026 [159].


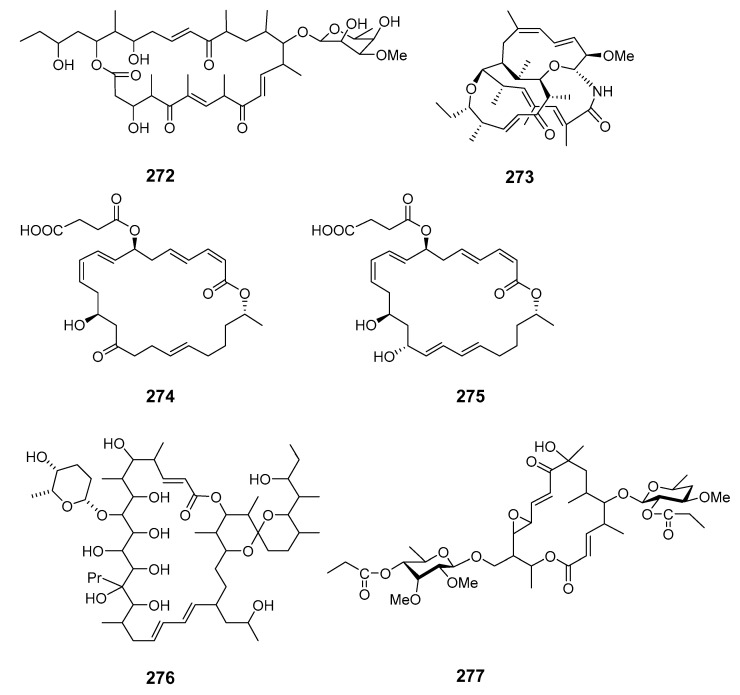


Cytotoxic macrolide IB-96212 (**276**) was obtained from marine actinomycete L-25-ES25-008 [160]. Chalcomycin B (**277**) was isolated from marine *Streptomycete* isolate B7064 and was bioactive in both microorganisms and microalgae [161]. Lobophorins A and B (**278**–**279**) have been extracted from culture broths of bacteria isolated from the surface of the Caribbean brown alga *Lobophora variegata* (Dictyotales) [162]. Micromonospolides A–C (**280**–**282**) were produced by *Micromonospora* sp. (*M.* sp.) and demonstrated inhibition of gastrulation in starfish embryos [163,164]. 


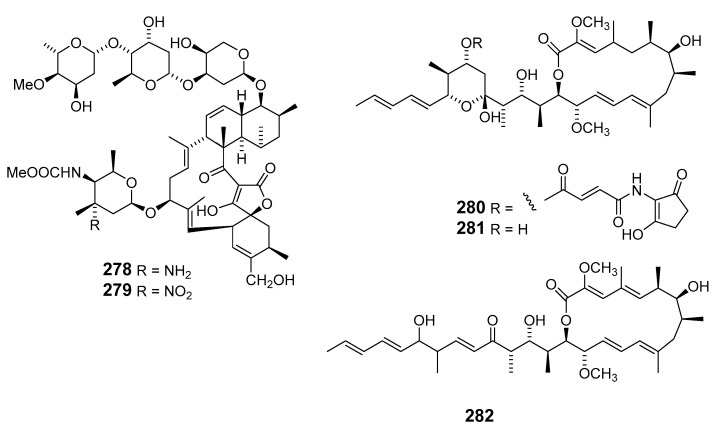


Marinomycins A–D (**283**–**286**) were isolated from actinomycete “*Marinispora*”. These marinomycins showed antibacterial activity towards methicillin-resistant *S. aureus* (MRSA), while marinomycin A inhibited vancomycin-resistant *S. faecium* (VREF) and *C. albicans* (weakly). Marinomycins A–C demonstrated cytotoxic activity against a panel of 60 tumor cell lines, including six of the eight melanoma cell lines [165]. 


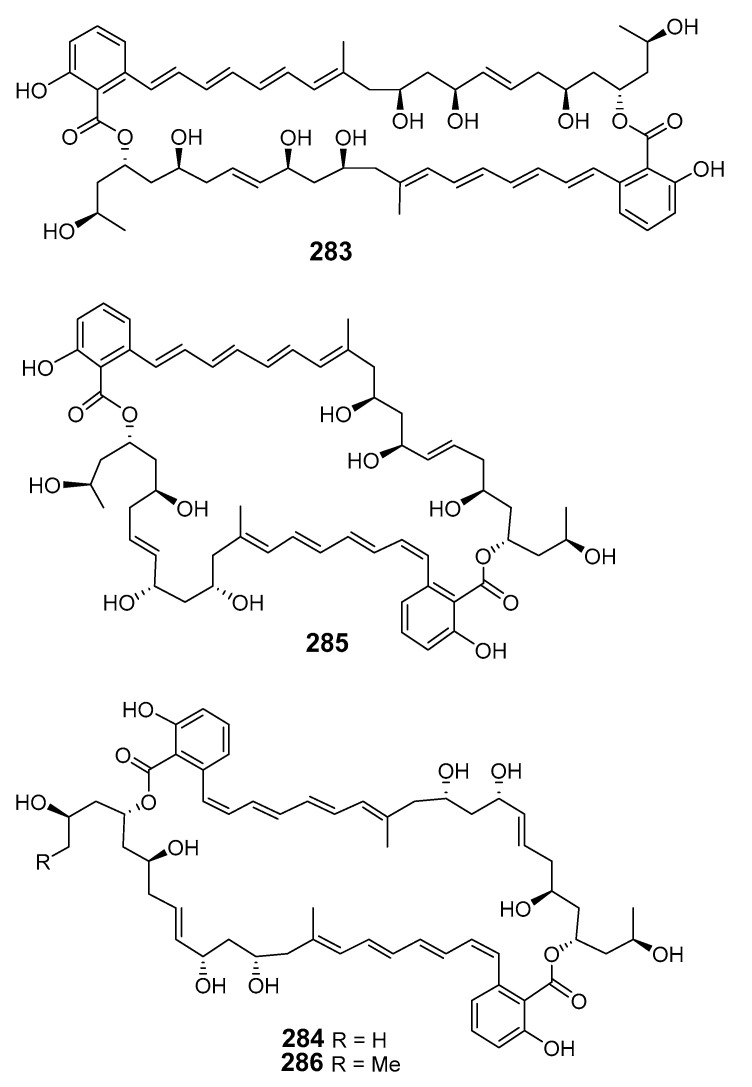


Marine actinomycete *Salinispora arenicola* yielded the three macrolide polyketides arenicolides A–C (**287**–**289**), with arenicolides A showing moderate cytotoxicity [166]. Macrolactin S (**290**) has been reported in a culture of marine *Bacillus* sp. [167]. The actinomycete strain CNQ-140 in the genus “*Marinispora*” yielded polyene macrolides marinisporolides A (**291**) and B (**292**), which photoisomerized to the geometric isomers marinisporolides C–E, suggesting that they may be artefacts [168]. *S. hygroscopicus* (associated with the marine fish *Halichoeres bleekeri*) produced halichoblelides B (**293**) and C (**294**), which are cytotoxic to tumor cells [169].


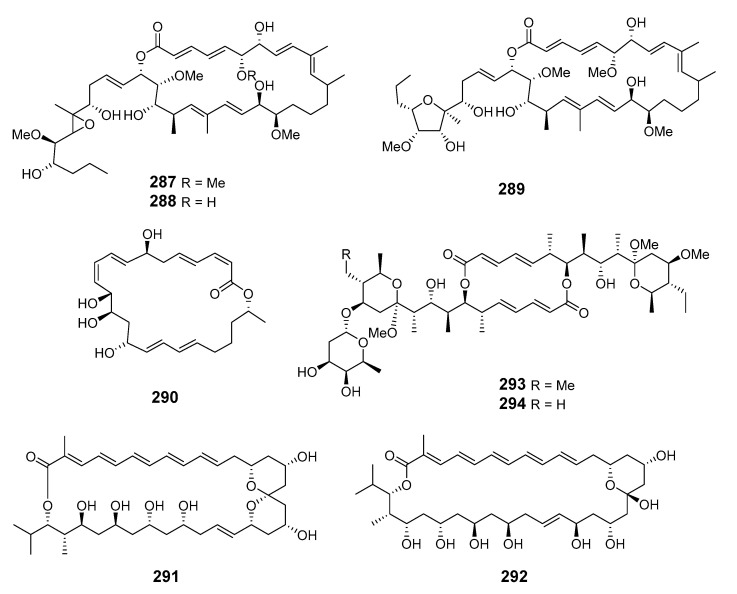


Two 36-membered macrolides, bahamaolides A and B (**295**–**296**), were obtained from the culture of a marine actinomycete *S.* sp. isolated from a sediment sample collected at North Cat Cay in the Bahamas [170]. 


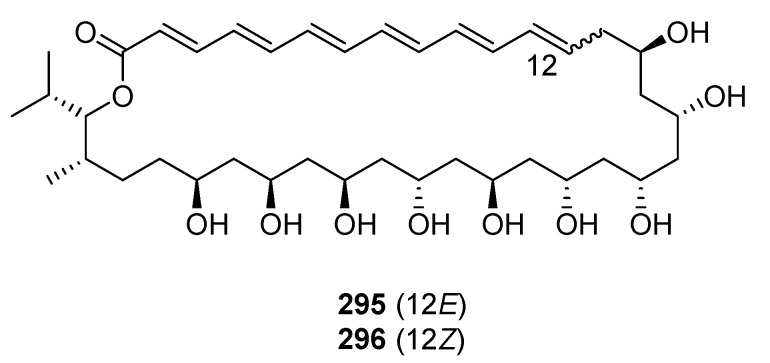


*B. subtilis* isolated from marine sediment collected at Gageocho (Republic of Korea) was a source of three new glycosylated methoxy-macrolactins (**297**–**299**) [171]. Three new 24-membered macrolactones, macrolactins X–Z (**300**–**302**), featuring an oxetane, an epoxide, and a tetrahydropyran ring, were isolated from an ethyl acetate extract of a marine *B.* sp. [172]. Cytotoxic juvenimicin C (**303**) was produced by a marine-derived actinomycete strain (CNJ-878) [173]. The *M.* strain FIM07-0019 isolated from shallow coastal waters near the island of Chiloe (Chile) produced a 20-membered macrolide, levantilide C (**304**) [174]. 


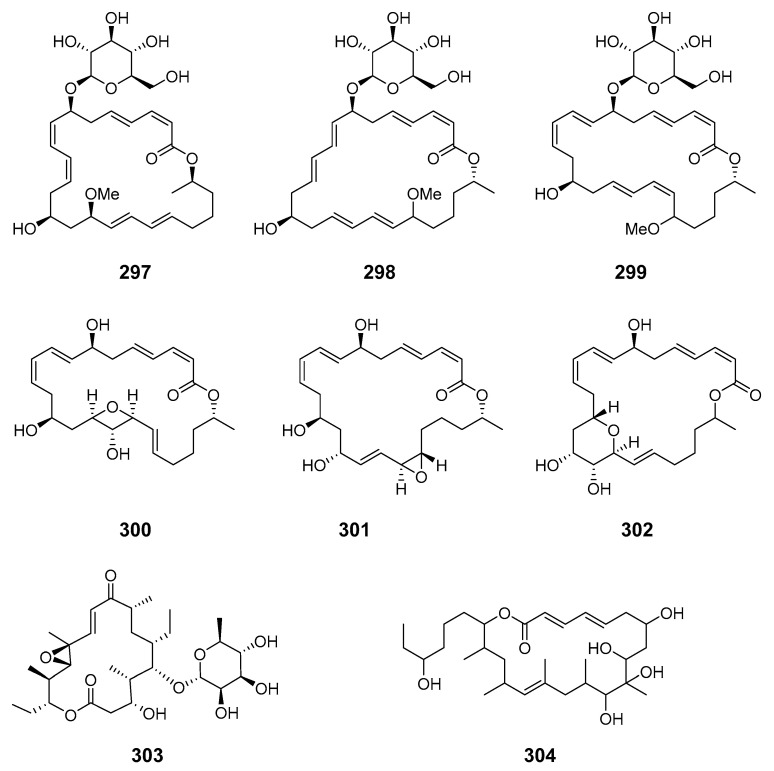


Investigation of a *S.* sp. in sediment from Heishijiao Bay (Dalian, China) yielded 11′,12′-dehydroelaiophylin (**305**) and 11,11′-O-dimethyl-14′-deethyl-14′-methylelaiophylin (**306**)—both 6-deoxyhexose-containing antibiotics—with the former exhibiting inhibition of MRSA and vancomycin-resistant *Enterococci* pathogens [175]. A rare 18-membered macrolide, macplocimine A (**307**), was produced by a marine-derived filamentous sulfur bacteria, *Thioploca* sp. [176]. A potent anthrax antibiotic, anthracimycin (**308**), was isolated from marine sediment-derived actinomycete *S.* sp. (Santa Barbara, California, U.S.A.) [177]. Fijiolides A (**309**) and B (**310**) were identified in marine-derived bacteria of the genus *Nocardiopsis* and demonstrated inhibition towards TNF-α-induced NFκB activation (fijiolide A to a greater extent than fijiolide B) [178]. Astolides A (**311**) and B (**312**) were obtained from *S. hygroscopicus* in the alkaline soil of the Saratov region of Russia. They exhibited significant cytotoxicity towards doxorubicin-resistant human leukemia cells [179]. Two hygrolidin macrolides, catenulisporidins A (**313**) and B (**314**), were isolated from the actinobacterium *Catenulispora* sp. KCB13F192 [180]. 


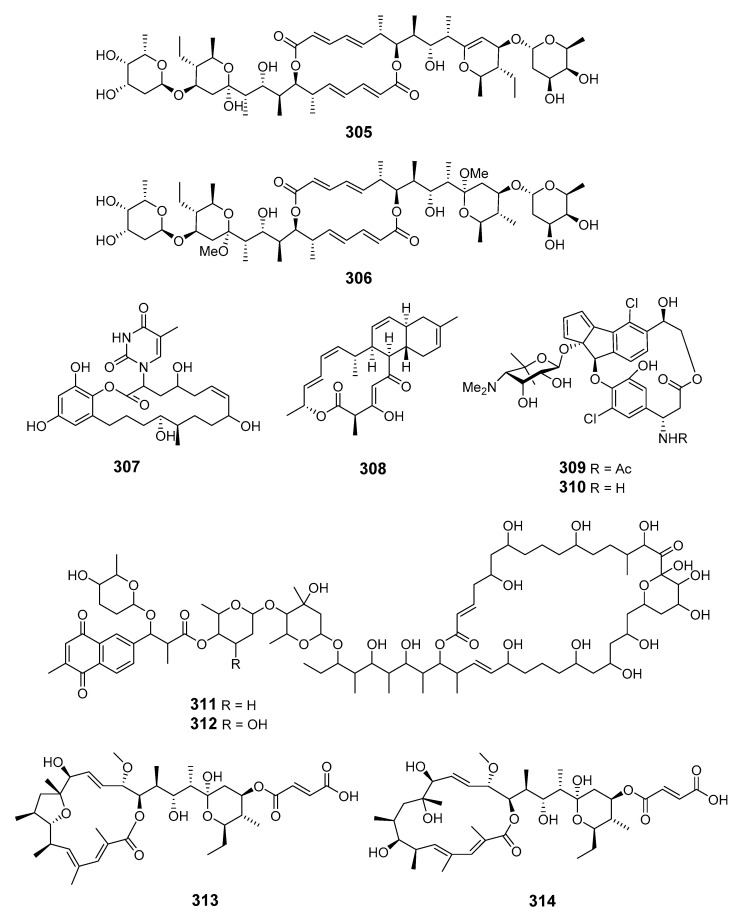


##### Cyanobacteria

Cyanobacteria *Scytonema mirabile* BY-8-1, *S. burmanicum* DO-4-1, and *S. ocellatum* DD-8-1, FF-65-1 and FF-66-3 have been reported to produce tolytoxin (**315**). *S. burmanicum* DO-4-1 also yielded scytophycin B (**316**), 6-hydroxyscytophycin B (**317**), 19-O-demethylscytophycin C (**318**), 6-hydroxy-7-O-methylscytophycin E (**319**), and scytophycin E (**320**) [181]. A macrolide, oscillatoriolide (**321**), was isolated from Japanese *Oscillatoria* sp. and demonstrated inhibition towards fertilized echinoderm eggs [182]. The marine cyanobacterium *Lyngbya bouillonii* (*L. bouillonii*) collected on Laing Island (Papua New Guinea) produced lyngbyaloside (**322**) [183] in addition to the macrolides laingolide (**323**), madangolide (**324**), and laingolide A (**325**) [184,185], and the glycosidic macrolide lyngbouilloside (**326**), for which the configuration of C-11 was later revised [186,187].


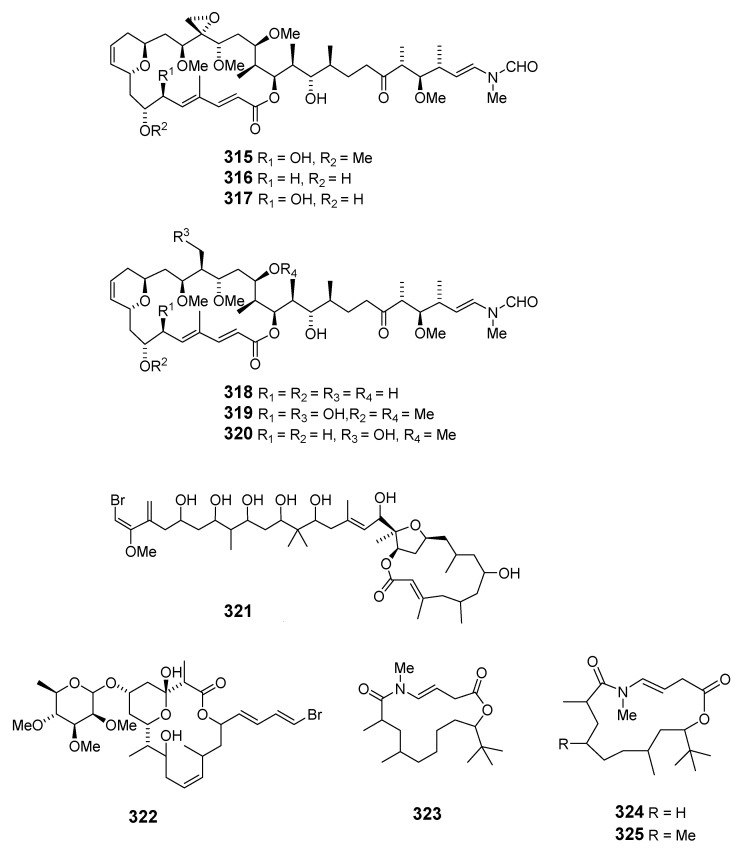


Two glycosylated swinholides, ankaraholides A (**327**) and B (**328**), together with swinholide A previously obtained from the marine sponge *T. swinhoei* [91], were isolated from cyanobacterium *Geitlerinema* sp. collected in Madagascar [188]. Cyanolide A (**329**), demonstrating significant molluscicidal activity towards the snail vector *Biomphalaria glabrata*, was also isolated from *L. bouillonii* from Papua New Guinea [189]. Biselyngbyolide A (**330**) was isolated from *L.* sp. and showed strong apoptosis-inducing activity in HeLa S3 and HL60 cells [190], while its analogs, biselyngbyolide B–D (**331**–**333**), were produced by another *L. cyanobacterium* sampled on Tokunoshima Island (Japan) [191]. Biselyngbyolide B exhibited inhibition and apoptosis-inducing activity in HeLa S3 and HL60 cells and increased the cytosolic Ca^2+^ concentration in HeLa S3 cells [191]. 


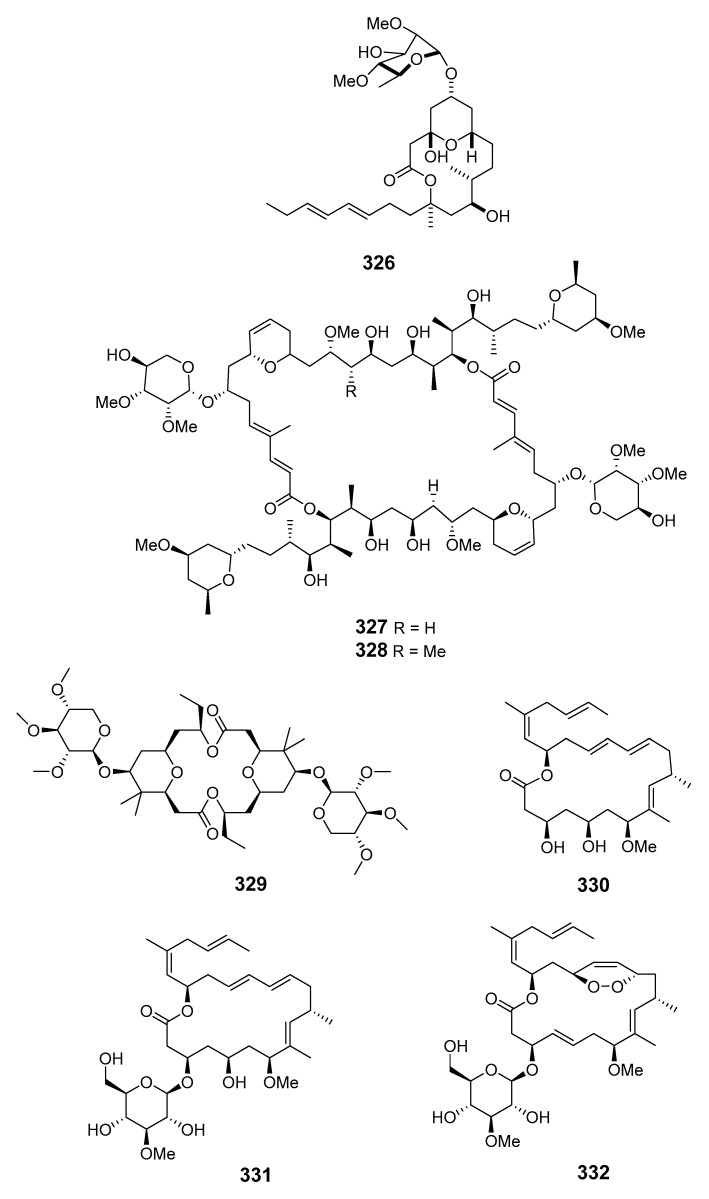



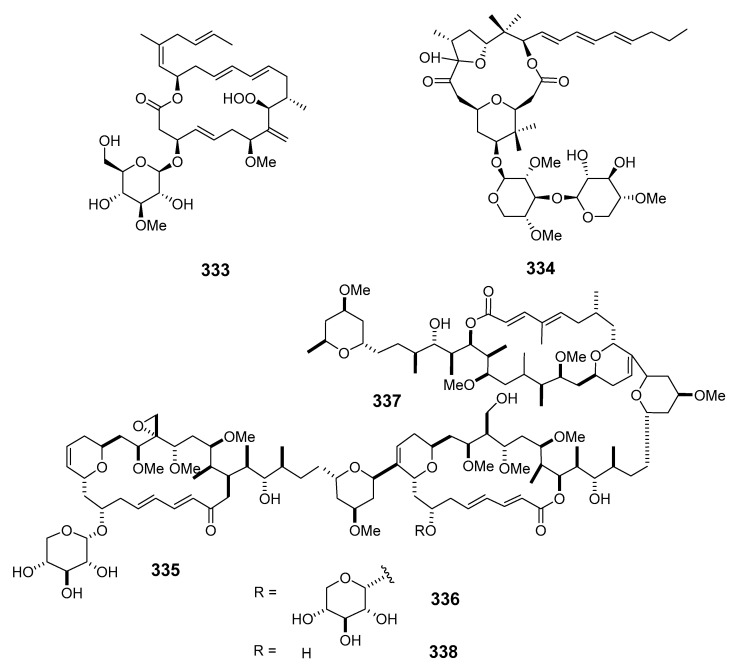


The Caribbean Okeania cyanobacterium VQR28MAR11-2 has been reported to produce polycavernoside D (**334**) [192], while four cytotoxic macrolides, leptolyngbyolides A–D (**335**–**338**), have been isolated from *Leptolyngbya* sp. collected in Okinawa [193].

##### Dinoflagellates

Amphidinolide E (**339**) was isolated from the Okinawan flatworm *Amphiscolops* sp. (*Amphis.* sp.) and exhibited cytotoxicity towards murine leukemia cells L1210 and L5178Y [194]. The absolute stereochemistry of amphidinolide E was determined by NMR spectroscopy, modified Mosher’s method and the exciton chirality method [195]. The potent cytotoxic macrolides amphidinolides F (**340**), G (**341**) and H (**342**) were produced by dinoflagellate *Amphidinium* sp. (*Amphid.* sp.) associated with the Okinawan flatworm *Amphis. breviviridis* [196,197]. 


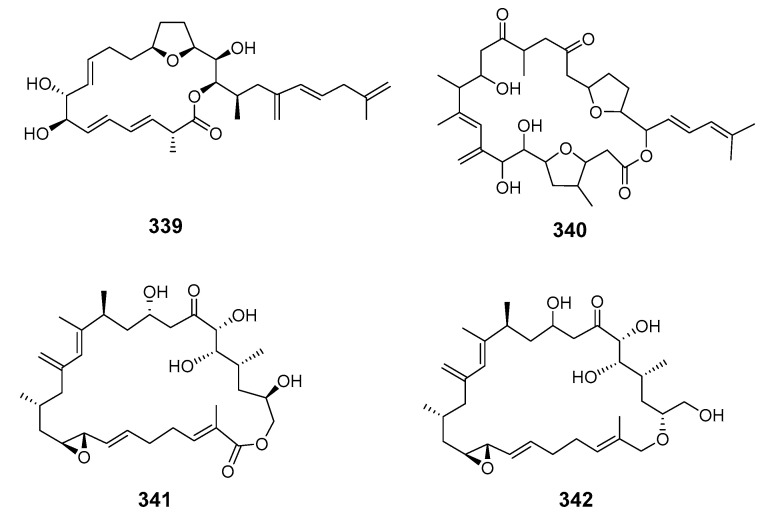


Amphidinolides G and H were elucidated by X-ray diffraction analysis and interconversion [198]. Amphidinolides J (**343**) and K (**344**) were isolated from symbiotic dinoflagellate *Amphid.* sp. and later synthesized [199,200]. Amphidinolides B1 (**345**), B2 (**346**) and B3 (**347**) were also isolated from *Amphid.* sp. [201,202,203], as were amphidinolides L (**348**), M (**349**) and N (**350**) [204,205,206]. 


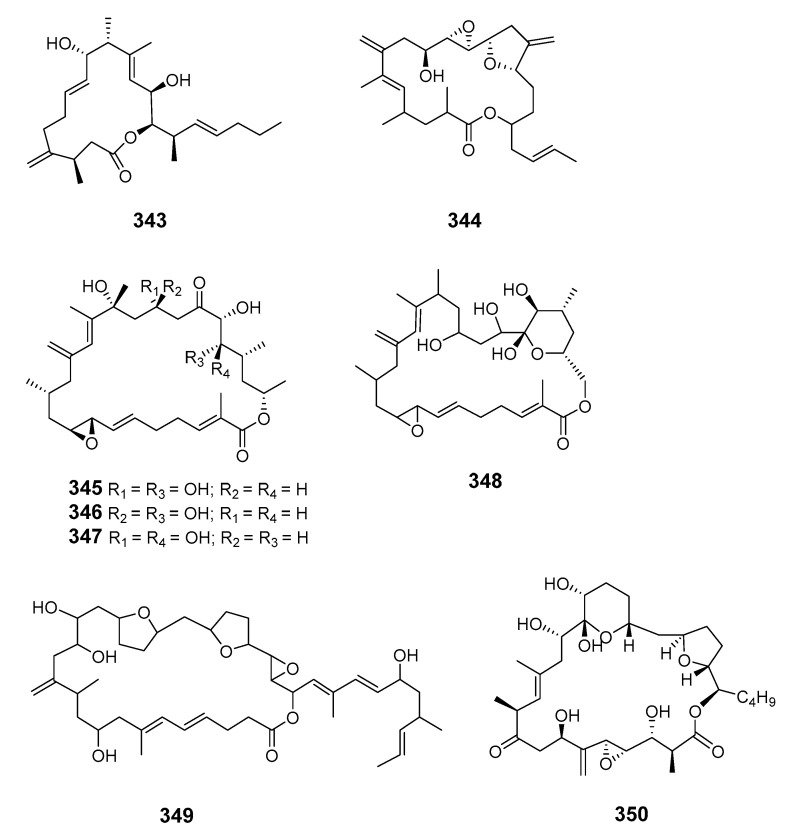


The structure of amphidinolide N was later revised and stereochemistry assigned [207]. Cytotoxic 15-membered macrolides, amphidinolides O (**351**) and P (**352**), were also isolated from *Amphis.* sp. [208]. The absolute stereochemistry of amphidinolide P was confirmed by convergent total synthesis [209]. The 12-membered macrolide amphidinolide Q (**353**), showing moderate cytotoxicity towards murine lymphoma L1210 cells in vitro (IC_50_ 6.4 μg/mL), was obtained from the symbiotic flatworm *Amphis.* sp. of dinoflagellate *Amphid*. sp. [210]. Amphidinolide Q was synthesized stereoselectively by combined Julia coupling, Myers alkylation, and Yamaguchi lactonization [211]. The absolute configurations at five chiral centers in amphidinolide Q were determined as 4*R*, 7*R*, 9*S*, 11*R*, and 13*R* on the basis of NMR analysis and a modified Mosher’s method [212]. Cytotoxic macrolides amphidinolides R (**354**) and S (**355**) were also isolated from *Amphid.* sp. [213]. The 20-membered macrolide amphidinolide U (**356**) was obtained from a cultured *Amphid.* sp. Y-56 isolated from the flatworm *Amphis.* sp. in Okinawa [214]. A 25-membered macrolide, amphidinolide C3 (**357**), was also obtained from the Y-56 dinoflagellate strain and exhibited cytotoxicity towards P388, L1210 and KB cells [215]. Y-56 has also been reported to yield the 19-membered macrolide amphidinolide T (T1) (**358**) [216], while the *A.* sp. Y-5 produced the 14-membered polyene amphidinolide V (**359**) [217]. Total synthesis of amphidinolide V was accomplished and the absolute stereochemistry assigned [218]. Analogs of amphidinolides T2 (**360**), T3 (**361**), T4 (**362**), and T5 (**363**) were produced by *Amphid.* sp. [219,220]. Amphidinolides H2 (**364**), H3 (**365**), H4 (**366**), H5 (**367**), G2 (**368**), and G3 (**369**) were produced by *Amphid.* sp. strain Y-42 isolated from marine acoel flatworms *Amphis.* sp. The absolute configurations of these compounds were determined by coupling constant data, distance geometry calculations, and chemical means [221]. Amphidinolide T2 was synthesized using methyl (*S*)-lactate via a 16-step linear sequence [222]. Amphidinolide W (**370**) was isolated from an *Amphid.* sp. and the absolute stereochemistry determined by a combination of *J*-based configuration analysis and modified Mosher’s method [223]. Total synthesis was later achieved and its C-6 stereochemistry revised [224]. Amphidinolides X (**371**) and Y (**372**) were produced by symbiotic dinoflagellate *Amphid.* sp. strain Y-42 from Okinawan *Amphis.* species. Amphidinolide Y exists as a 9:1 equilibrium mixture of the 6-keto- and 6(9)-hemiacetal forms (**373**). Both amphidinolides X and Y showed significant cytotoxicity against murine lymphoma L1210 and human epidermoid carcinoma KB cells in vitro [225,226]. Two 26-membered macrolides, amphidinolides B6 (**374**) and B7 (**375**), were isolated from a culture of a symbiotic dinoflagellate *Amphid.* sp. from *Amphis.* sp. and demonstrated cytotoxicity against human B lymphocyte DG-75 cells [227]. Amphidinolide C2 (**376**) was isolated from dinoflagellate *Amphid.* sp. (Y-71 strain) [228]. 


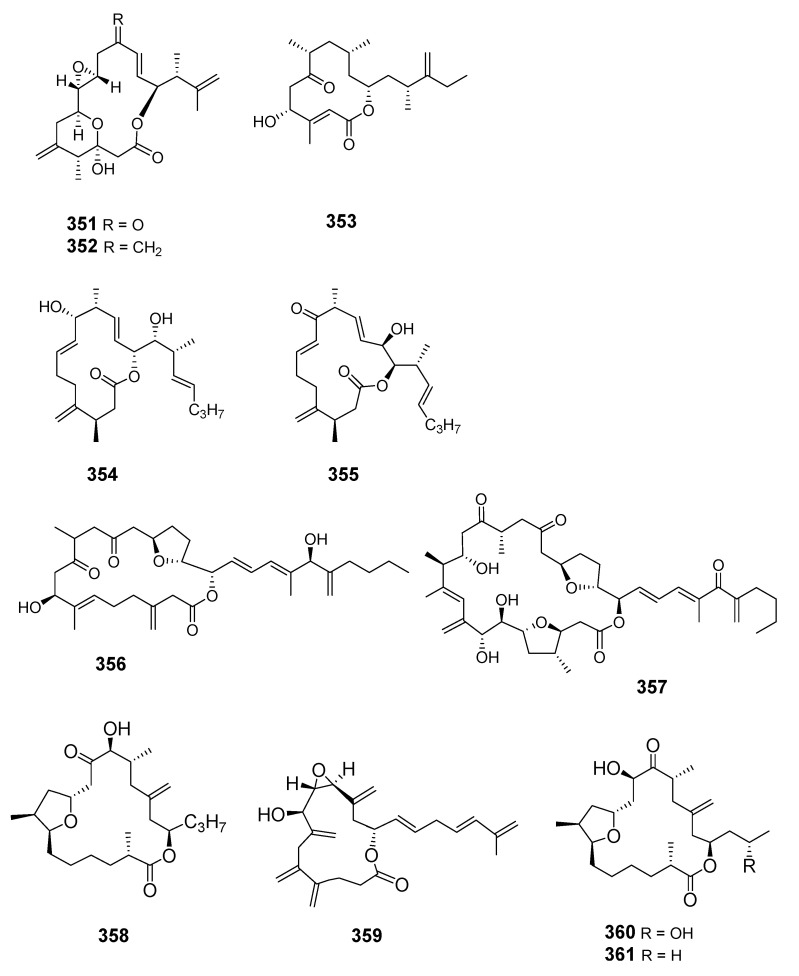



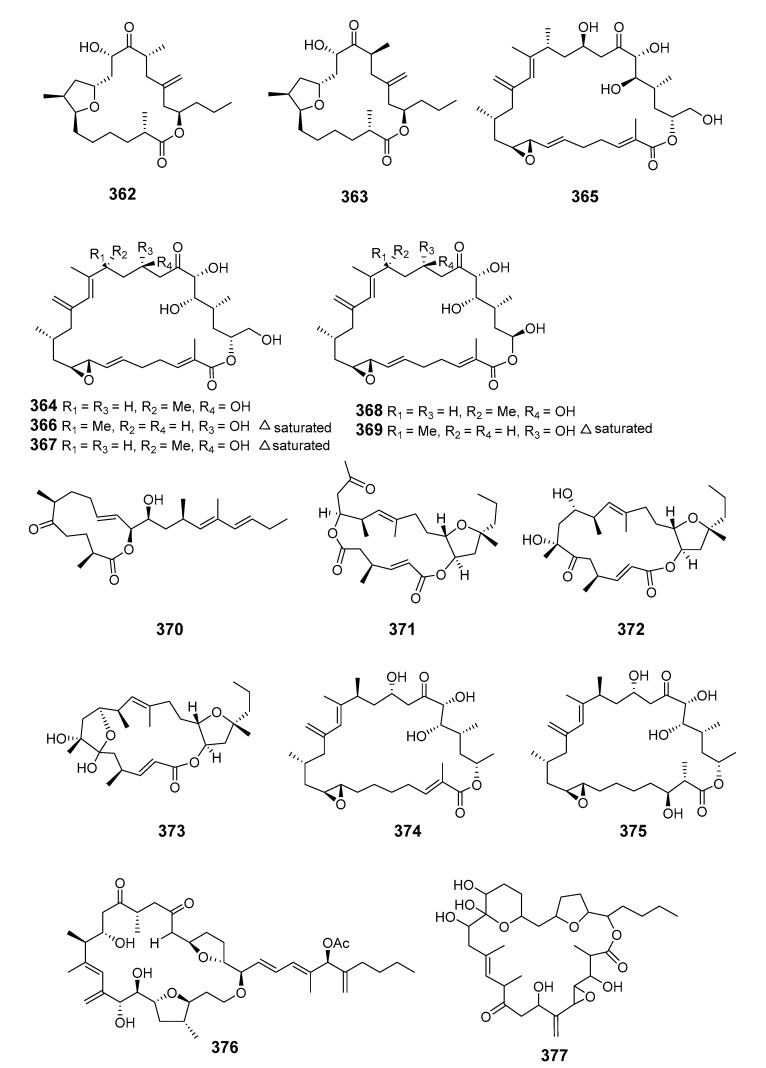


The *Amphid.* strain S1-36-5 yielded the highly cytotoxic 26-membered caribenolide I (**377**) [229].

The 13-membered macrolide amphidinolactone A (**378**) and a 26-membered macrolide amphidinolactone B (**379**) have been isolated from cultures of *Amphid.* sp. Amphidinolactone A was synthesized totally via a ring-closing metathesis reaction and the absolute configuration was elucidated [8,230,231]. The vasoconstrictors zooxanthellatoxins A (**380**) and B (**381**) were isolated from a symbiotic dinoflagellate *Symbiodinium* sp. (Y-6 strain), which was associated with *Amphis.* sp. [232,233]. Bioassay-guided fractionation of a butanol extract of the tropical dinoflagellate *Prorocentrum maculosum* Faust yielded the fast-acting toxin prorocentrolide B (**382**) [234]. Hoffmanniolide (**383**) was identified in the marine dinoflagellate *P. hoffmannianum* [235]. The 20-membered iriomoteolide-1a (**384**), -1b (**385**) and -1c (**386**) were isolated from a marine benthic dinoflagellate *Amphid.* sp. (strain HYA024) [236,237]. 


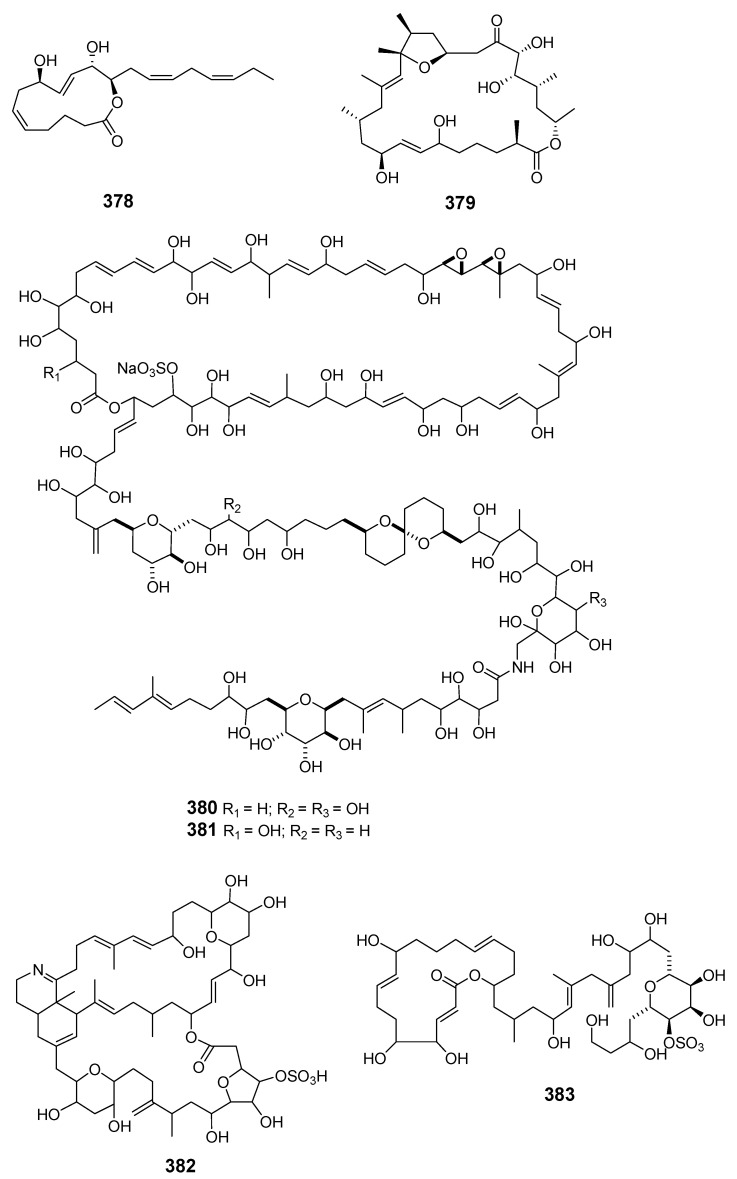



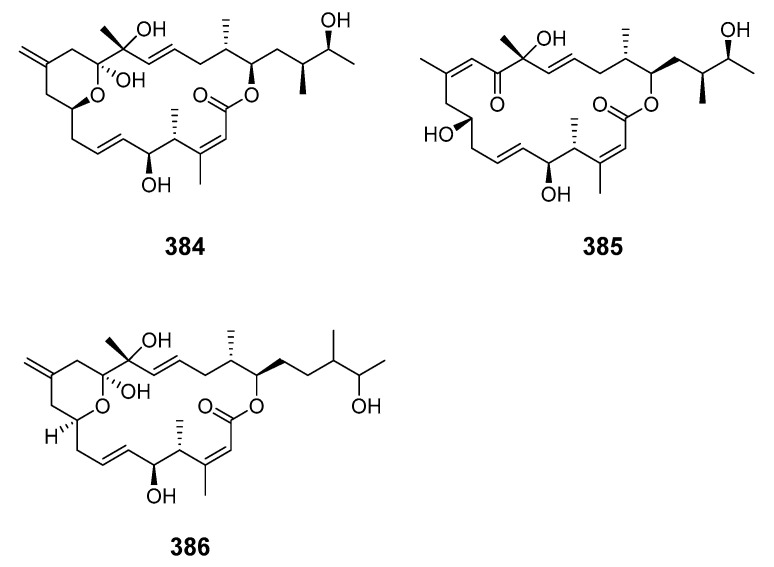


The cytotoxic 23-membered iriomoteolide-2a (**387**) was also obtained from *Amphid.* sp. [238]. The 15-membered macrolide iriomoteolide-3a (**388**) containing an allyl epoxide was obtained from *Amphid.* sp. strain HYA024 and was potently cytotoxic to human B lymphocyte DG-75 cells and Epstein–Barr virus (EBV)-infected Raji cells [239]. Iriomoteolide-4a (**389**) and -5a (**390**) were isolated from a benthic dinoflagellate *Amphid.* sp. (strain HYA024) and showed moderate cytotoxicity towards human B lymphocytes DG-75 [240]. The 15- and 19-membered iriomoteolide-9a (**391**) and -11a (**392**) were cytotoxic towards human cervix adenocarcinoma HeLa and murine hepatocellular carcinoma MH134 cells [241].


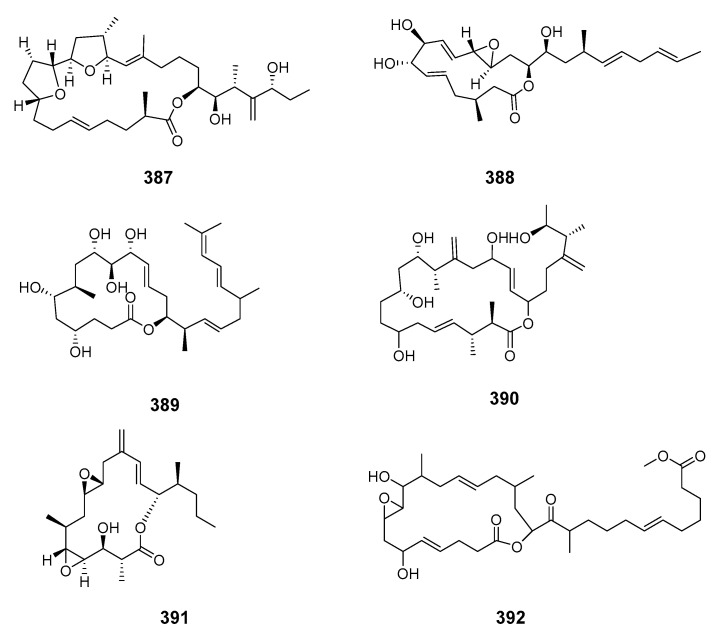


Iriomoteolide-10a (**393**) and -12a (**394**) were isolated from a marine dinoflagellate *Amphid.* sp. (KCA09053 strain) with iriomoteolide-10a being cytotoxic to human cervix adenocarcinoma HeLa and murine hepatocellular carcinoma MH134 cells [242]. The 62-membered novel polyol macrolide symbiodinolide (**395**) was isolated from the symbiotic dinoflagellate *Symbiodinium* sp. (*S*. sp.) and showed significant voltage-dependent N-type Ca^2+^ channel-opening activity at 7 nM and immediately ruptured the surface tissue of the acoel flatworm *Amphis.* sp. at 2.5 mM [14]. The stereochemistries of C-23–C-34 were revised by stereoselective synthesis and the (17*S*,18*R*,21*R*) configurations were determined by synthesis [243,244]. The synthesis of the C-33–C-42 fragment elucidated (36*S*,40*S*) and (C-1′–C-25′) [243,244,245]. The dinoflagellate-derived macrolide acuminolide A (**396**) caused potent stimulation of actomyosin ATPase activity [246]. The 25-membered polyketide-derived macrocycle belizentrin (**397**) was isolated from cultures of the marine dinoflagellate *Prorocentrum belizeanum* [247]. Gymnodimine D (**398**) was extracted and purified from a culture of dinoflagellate *Alexandrium ostenfeldii* from the Baltic Sea [248]. Symbiodinolactone A (**399**) was isolated from a culture of the symbiotic marine dinoflagellate *S.* sp. [249].


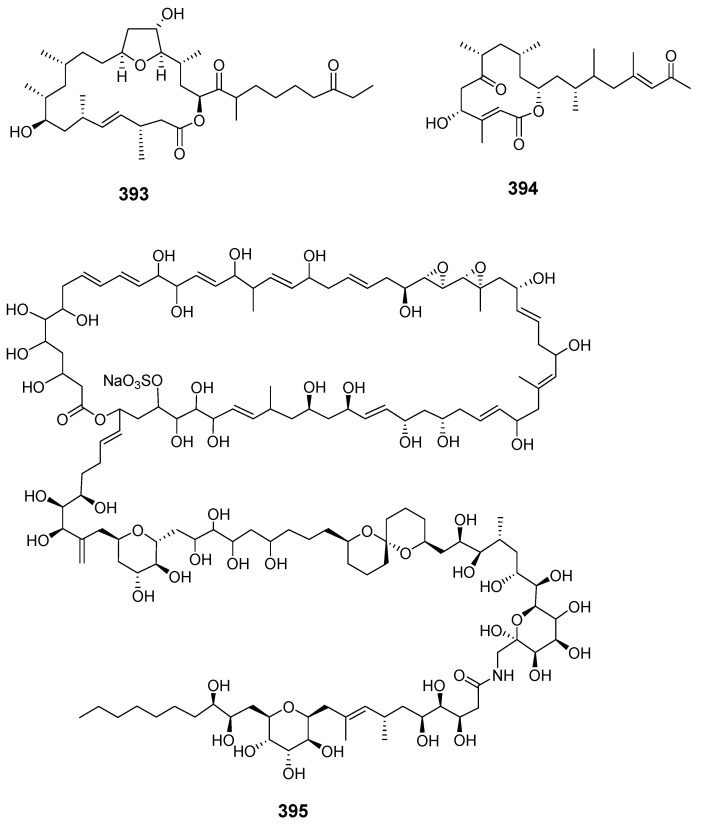



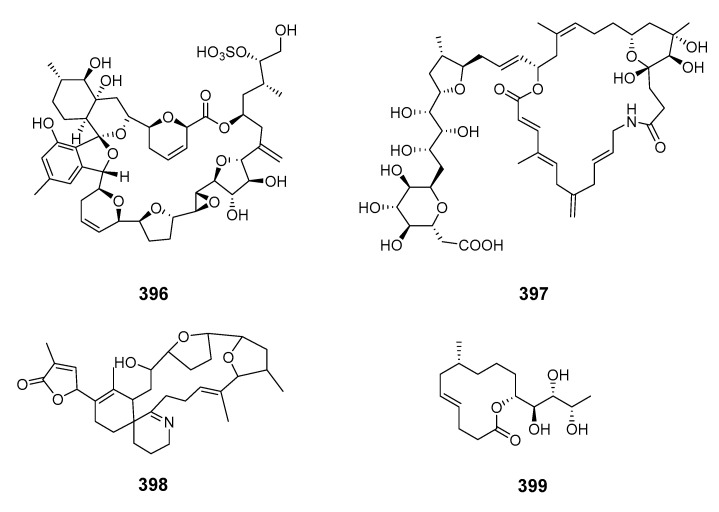


#### 2.1.3. Red algae

*Polycavernosa tsudai* (*Gracilaria edulis*) contained the macrolide polycavernoside A (**400**), which led to human illness and death in Guam [250]. The relative configuration of polycavernosolide A was assigned and the sugar substructure was synthesized [251,252]. Its structure was confirmed by total synthesis in a stereocontrolled manner [253]. Polycavernosides A2 (**401**), A3 (**402**), B (**403**) and B2 (**404**) were also obtained from *Polycavernosa* red algae [254]. 


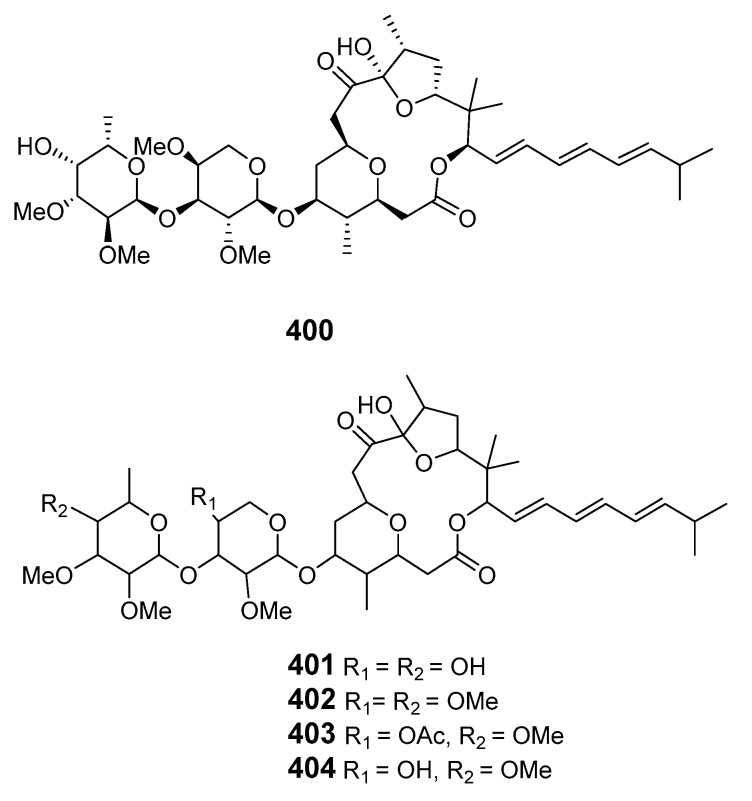


Two analogs of polycavernosolide A, polycavernosides C (**405**) and C2 (**406**), were isolated from the red alga *Gracilaria edulis* (*G. edulis*) [255]. Manauealides A–C (**407**–**409**) were isolated from extracts of red alga *G. coronopifolia* [256]. Anhydrodebromoaplysiatoxin (**410**) and manauealide C were extracted from Hawaiian *G. coronopifolia* [257]. Investigation of Fijian red alga *Callophycus serratus* (*C. serratus*) led to the isolation of three diterpene-benzoate natural products: bromophycolides A (**411**) and B (**412**), and a nonhalogenated compound (**413**). Bromophycolides A and B exhibited moderate antibacterial and antifungal properties while bromophycolides A demonstrated potent anti-HIV and moderate cytotoxic activities [258]. Bromophycolides C–I (**414**–**420**) were also isolated from extracts of *C. serratus*. All the bromophycolides exhibited modest antineoplastic activity towards a range of human tumor cell lines while bromophycolides F and I showed weak antifungal activity [259]. 


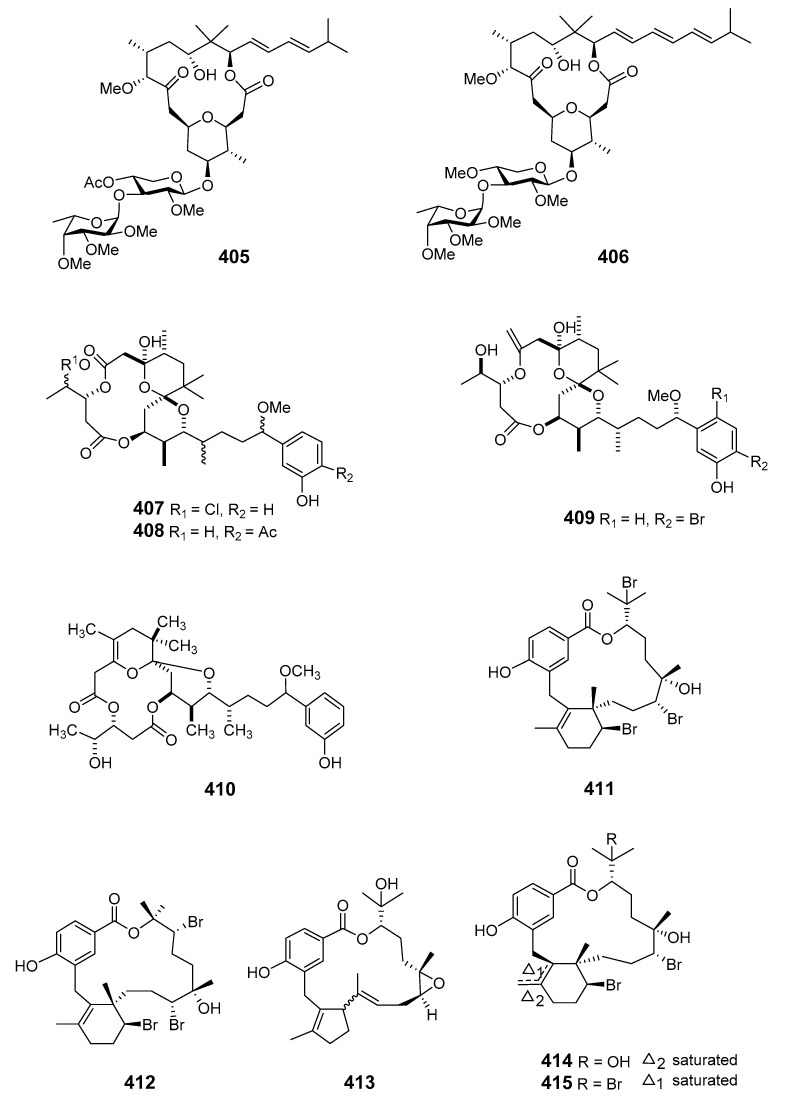



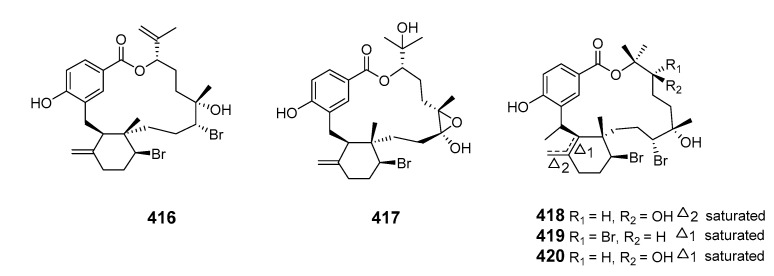


Further investigation of the *C. serratus* extract yielded a series of unusual antimalarial diterpene-benzoate macrolides, bromophycolides J–Q (**421**–**428**), with a range of moderate to strong antimicrobial and anticancer properties [260]. *C. serratus* was also a source of the diterpene-benzoate macrolides bromophycolides R–U (**429**–**432**). These demonstrated modest cytotoxicity toward selected human cancer cell lines while bromophycolide S was active (at submicromolar concentrations) against the human malaria parasite *Plasmodium falciparum* (*Pla. falciparum*) [261]. 


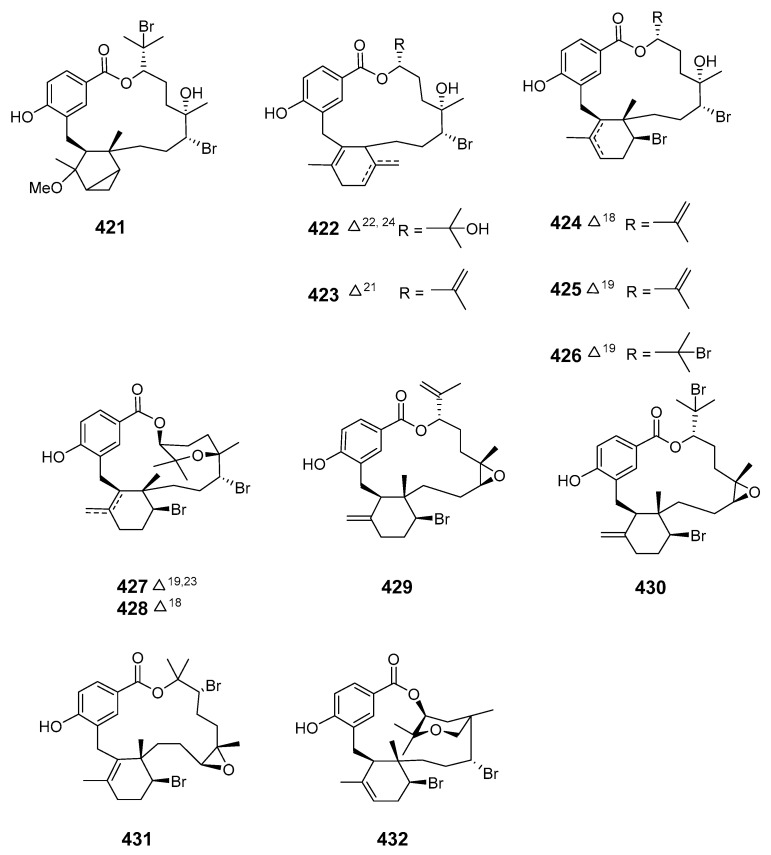


The α-pyrone macrolides neurymenolides A (**433**) and B (**434**) were obtained from the Fijian red alga *Neurymenia fraxinifolia* [262]. The brown alga *Ecklonia stolonifera* produced ecklonialactones C (**435**) and D (**436**) containing a 14-membered lactone moiety, and ecklonialactones E (**437**) and F (**438**), with a 16-membered moiety [263]. The absolute configurations of ecklonialactones A, B and E were determined from chiroptical data [264]. Eight oxylipins (**439**–**446**) with a macrolide scaffold and one cymathere-type oxylipin with an open ring were isolated from the brown alga *Eisenia bicyclis*. The absolute configurations of compounds **439**–**443** and **446** were determined by NMR spectroscopy with the relative stereochemistry at C-9 in **446** remaining unassigned [265]. The metamorphosis-enhancing macrodiolide luminaolide (**447**) was isolated from the crustose coralline alga *Hydrolithon reinboldii* and its absolute relative configuration was determined by NMR spectroscopy with the relationships of the two side chains to the macrolide ring remaining unassigned [266,267].


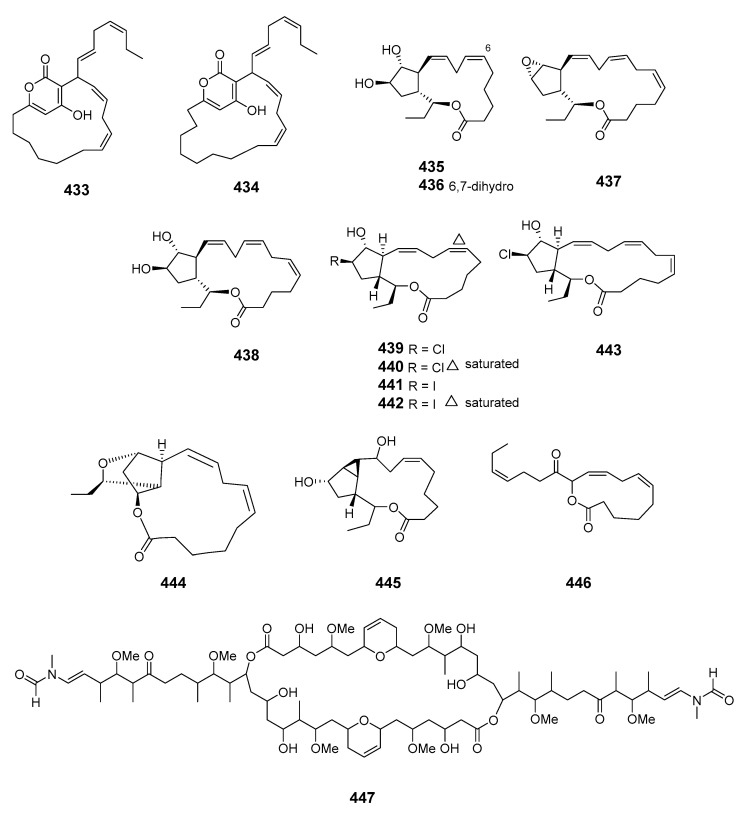


#### 2.1.4. Cnidarians

Two avermectin congeners, avermectins B1c (**448**) and B1e (**449**), exhibiting moderate antifouling activity were obtained from *Anthrogorgia caerulea* collected in the South China Sea [268].


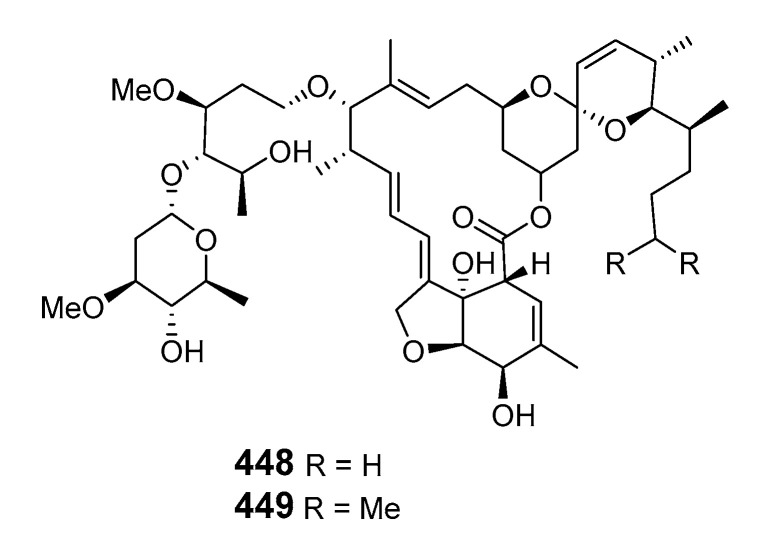


#### 2.1.5. Bryozoans

Large-scale isolation of bryostatin 1 (**450**) from the marine bryozoan *Bugula neritina* (L.) was carried out to provide material for clinical study [269]. Bryostatin 2 (**451**) has been converted to bryostatin 1 and bryostatin 12 (**452**) by selective protection and deprotection involving the C-26 hydroxyl group [270]. The stereochemistries of bryostatins 1 and 2 were assigned by X-ray analysis of *p*-bromobenzoate (**453**) [271], while the assignments of bryostatin 1 from ^1^H- and ^13^C-NMR were later revised [272]. Bryostatin 3 was isolated from *B. neritina* and reinvestigation of 2D NMR spectroscopic data revised the structure of bryostatin 3 to structure **454** [273].


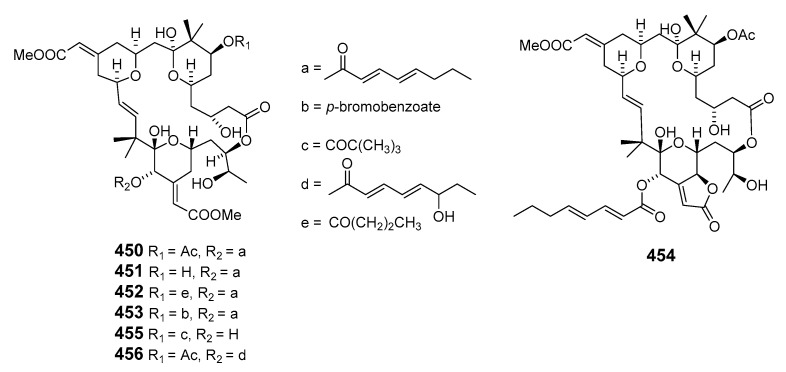


Further investigation of *B. neritina* led to the identification of bryostatins 14 (**455**) and 15 (**456**) [274]. The structures of bryostatin 3 and 20-epi-bryostatin 3 (**457**) have been elucidated by NMR spectroscopy [271,273,275]. Bryostatin 3 was then synthesized in an enantioselective manner [276]. Bryostatin 10 (**458**) was determined to be the major cytotoxic component of *B. neritina* [277]. Three additional antileukemic macrolides, bryostatins 16 (**459**), 17 (**460**), and 18 (**461**), were isolated in trace amounts from *B. neritina* from the Gulf of Mexico [278]. Antineoplastic bryostatin 19 (**462**) was isolated from *B. neritina* collected from the South China Sea [279]. A further member of the bryostatins, bryostatin 20 (**463**), was produced by the larvae of *B. neritina* and its structure determined by spectral comparison with previously described bryostatins [280]. Bioassay-guided isolation elucidated the first member of a new family of macrocycles, neristatin 1 (**464**), which was cytotoxic towards the P388 lymphocytic leukemia cell line [281].


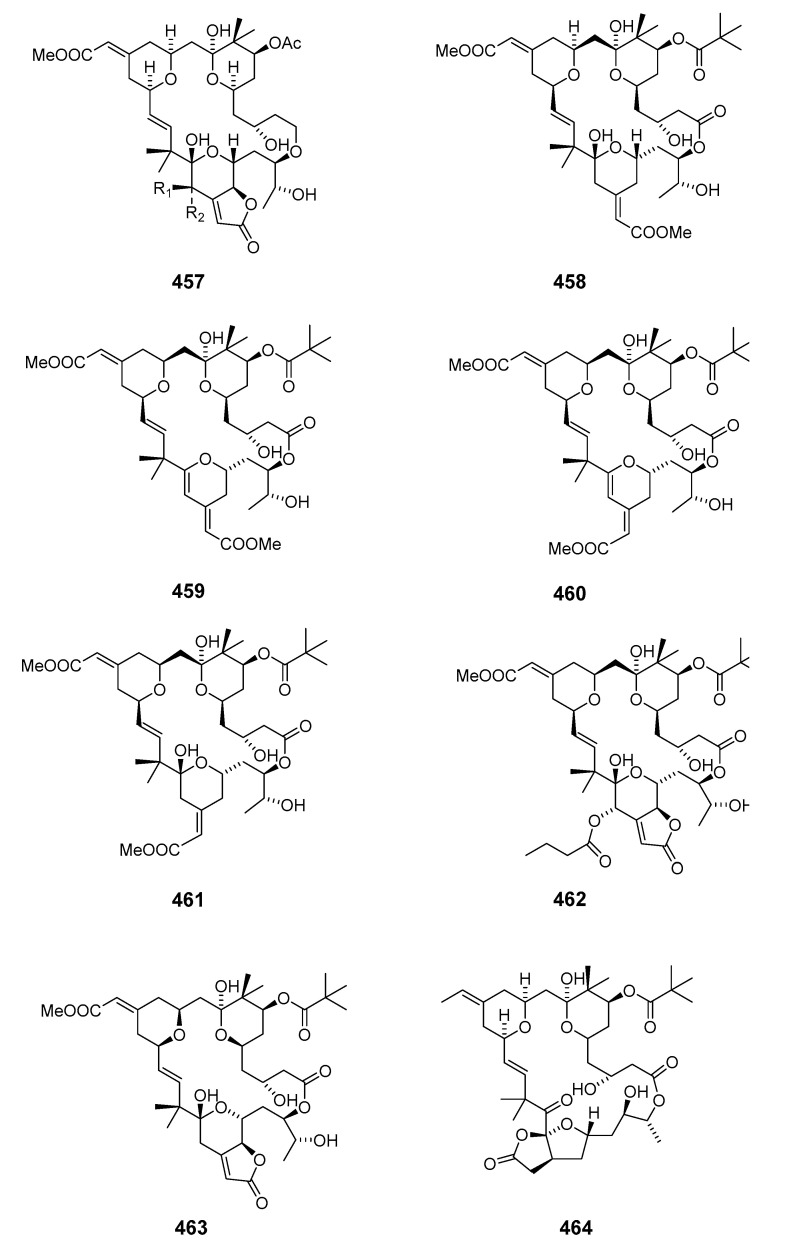


#### 2.1.6. Mollusks

Aplysiatoxin (**465**) was isolated from an extract of the sea hare *Stylocheilus longicauda* and synthesized [282,283]. The sea hare *Aplysia kurodai Baba* contained the novel and potently cytotoxic macrolides aplyronines A (**466**), B (**467**) and C (**468**). The absolute configuration of aplyronine A was assigned following enantioselective synthesis of its degradation products and total synthesis was also reported [284,285]. Five cytotoxic macrolides, aplyronines D–H (**469**–**473**), were also isolated from the Japanese sea hare *Aplysia kurodai* [286]. The 22-membered macrolide dolabelide A (**474**) and the diacetyl derivative dolabelide B (**475**), both cytotoxins, were obtained from the Japanese sea hare *Dolobella auricularia* [287]. Cytotoxic 24-membered macrolides dolabelides C (**476**) and D (**477**) were also isolated from *Dolabella auricularia*, the originally assigned structure of dolabelide D being confirmed by total synthesis [288,289]. Five unprecedented C-16 and C-18 fatty acid lactones named aplyolides A–E (**478**–**482**) were found in the skin of the marine mollusk *Aplysia depilans*, and were ichthyotoxic to the mosquito fish *Gambusia affinis* [290]. The stereochemistry of (-)-aplyolide A was confirmed by synthesis [291] and the absolute stereochemistries of aplyolides B–E were confirmed by total synthesis [292,293]. Pectenotoxins 4 (**483**) and 7 (**484**) were isolated from *Patinopecten yessoensis* scallops [294]. LC–MS analysis of shellfish extracts identified PTX-12 (**485**) as a pectenotoxin accumulating in Norwegian blue mussels (*Mytilus edulis*) and cockles (*Cerastoderma edule*) [295]. Dolastatin 19 (**486**), containing a 14-membered macrocyclic lactone linked to a 2,4-di-*O*-methyl-L-*R*-rhamnopyranoside, was found in the Gulf of California in the shell-less mollusk *Dolabella auricularia* [296]. The stereochemistry of (+)-dolastatin 19 was confirmed by total synthesis [297].


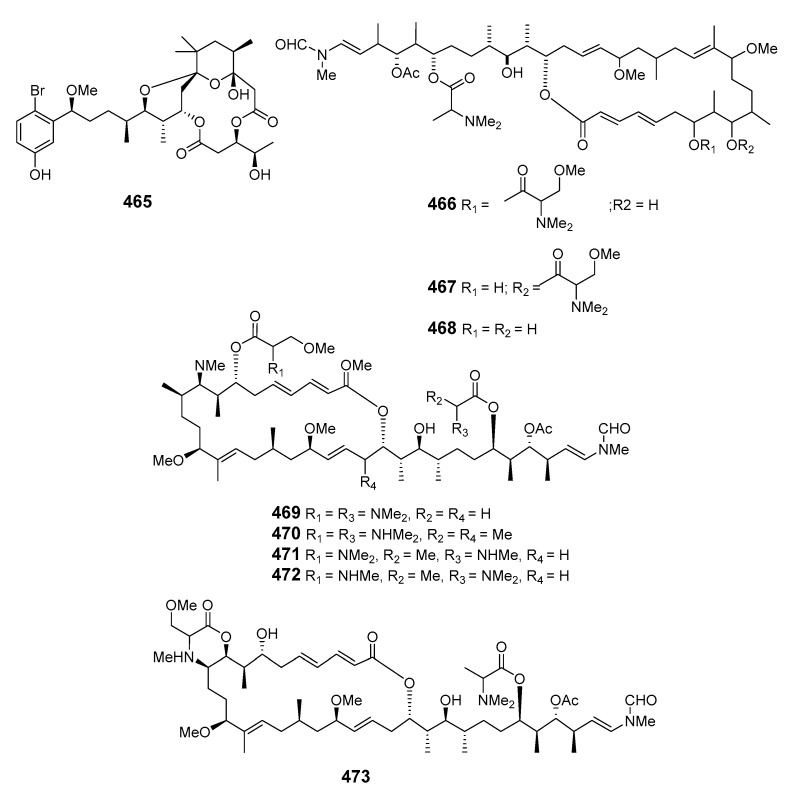


#### 2.1.7. Tunicates

Two 24-membered macrolide sulfates showing antineoplastic activity, iejimalides C (**487**) and D (**488**), were isolated from the Okinawan tunicate *Eudistoma* cf. *rigida* [298]. Two cytotoxic macrolides, lobatamides A (**489**) and B (**490**), were reported in the tunicate *Aplidium lobatum* [299]. *A. lobatum* from shallow waters in Australia, *A.* sp. from deep water, and an unidentified Philippine ascidian have been reported as sources of a series of macrolides, lobatamides C–F (**491**–**494**), demonstrating cytotoxicity towards human tumor cell lines [300]. The absolute stereochemistry of lobatamide C was determined by stereospecific synthesis [301]. The chlorinated macrolide haterumalide B (**495**) was obtained from an Okinawan ascidian *L.* sp. by bioassay-guided isolation and was shown to inhibit the first cleavage of fertilized sea urchin eggs at 0.01 μg/mL [302]. The Okinawan ascidian *Didemnidae* sp. was the source of the macrolides biselides A (**496**) and B (**497**) [303]. Further investigation of the *D.* sp. led to the isolation of biselides C (**498**), D (**499**) and E (**500**) which exhibited cytotoxicity against human cancer cells NCI-H460 and MDA-MB-231 [304]. Cytotoxic palmerolide A (**501**) was obtained from the Antarctic tunicate *Synoicum adareanum* [305] and its stereochemistry was revised and confirmed by synthesis [306,307]. 


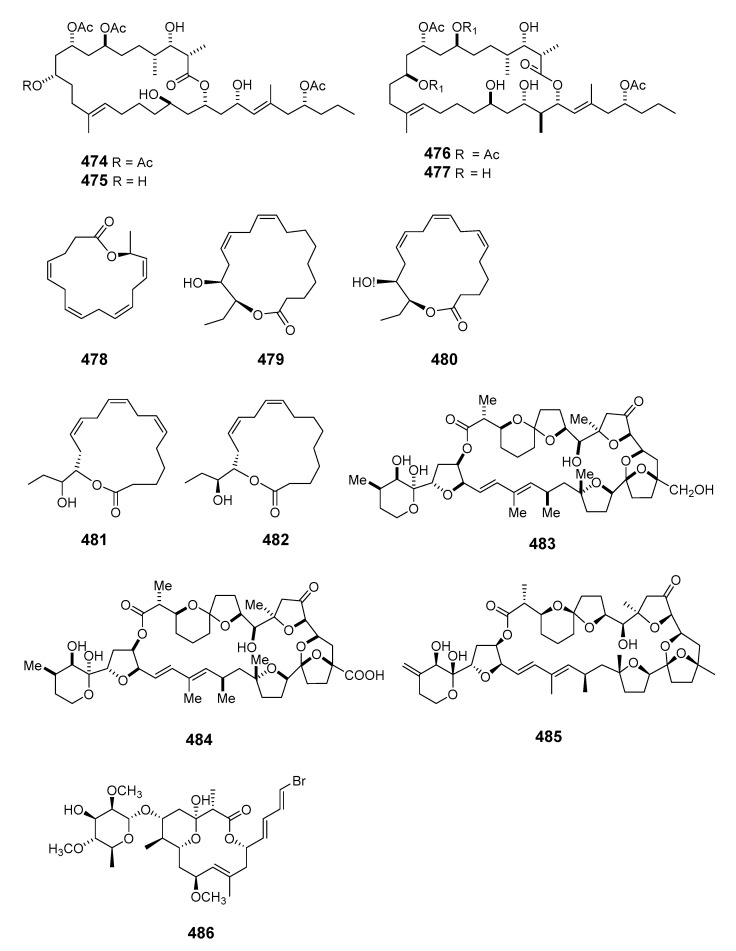



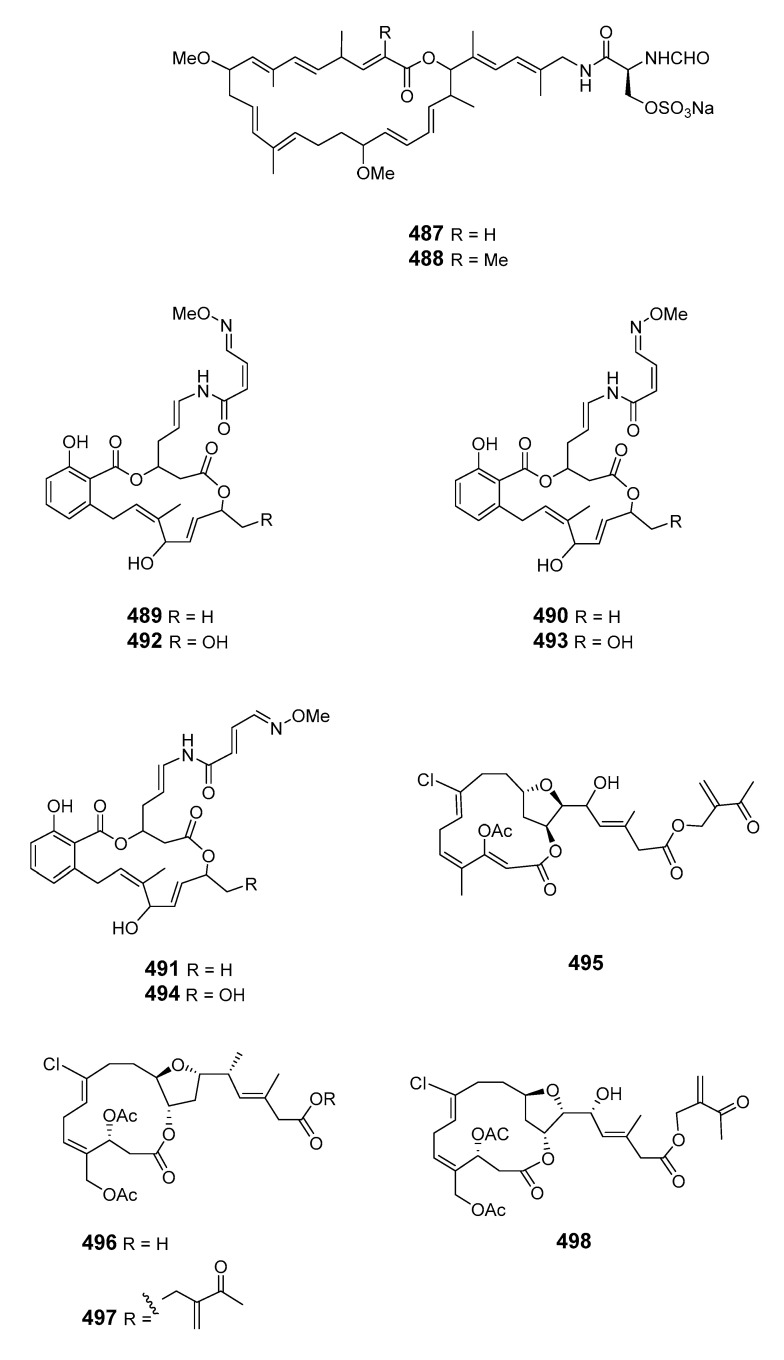



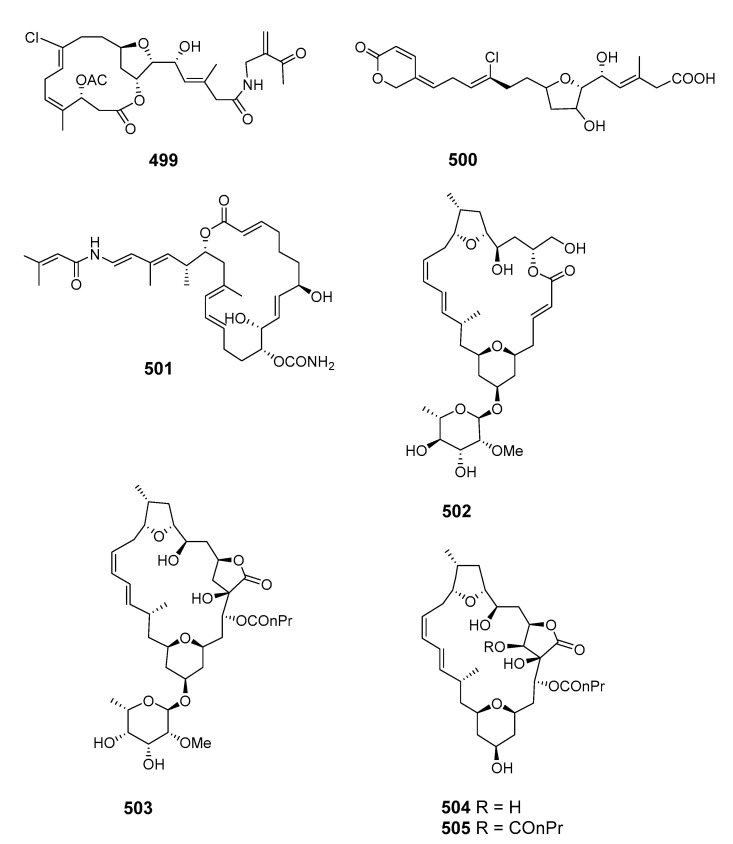


Glycosylated macrolides mandelalides A−D (**502**–**505**) were isolated from *Lissoclinum ascidian* collected in Algoa Bay near Port Elizabeth and the surrounding Nelson Mandela Metropole in South Africa [308].

### 2.2. Bioactivities of Marine-Derived Macrolides

The biological activities of marine-derived macrolides have been studied extensively. As listed in Table 1, marine macrolides harbor a broad range of bioactive properties including cytotoxicity, antibacteria, antifungi, antimitotic, antiviral, and other activities, with cytotoxicity being their most significant bioactivity.

**Table 1:** Biological activities of marine-derived macrolides.

Drug Class	Compounds	Pharmacology	Activities	Ref.
Cytotoxic ^a^	swinholides A–C (**1**–**3**)	KB cells	IC_50_: 0.041, 0,052, 1.1 μg/mL	[16]
	miyakolide (**13**)	P388 cells	IC_50_: 17.5 μg/mL	[21]
	spongiastatin 1 (**18**)	HL-60, NCI-116, DMS 114 et al.	GI_50_: 2.5–3.5 × 10^−11^ M	[26]
	dictyostatin 1 (**33**)	P388 cells	undetermined	[32]
	superstolide B (**37**)	KB, P388, NSCLC-N6-L16 cells	IC_50_: 0.005, 0.003, 0.039 μg/mL	[38]
	lasonolide A (**38**)	A-549, P388 cells	IC_50_: 40, 2 ng/mL	[40]
	latrunculin S (**44**), neolaulimalide (**45**)	P388, A549, HT29, MEL28 cells	IC_50_: 0.5–1.2 μg/mL, IC_50_: 0.01–0.05 μg/mL	[46]
	leucascandrolide A (**48**)	KB, P388 cells	undetermined	[48]
	altohyrtins B–C (**51**–**52**) 5-desacetylaltohytrin A (**53**)	KB cell;L1210 cells	IC_50_: 0.02, 0.4; 0.3 ng/mL; IC_50_: 0.03, 1.3, 2.3 ng/mL	[53]
	swinholide H (**54**)	P388 cells	undetermined	[13]
	neonorhalichondrin B (**55**), neohomohalichondrin B (**56**), 55-methoxyisohomohalichon-drin (**57**), 53-methoxyneoisohomohalichondrin B (**58a**)	P388 cells	IC_50_: 0.4, 0.8, 10, 0.1 ng/mL	[55]
	salicylihalamides A (**59**), B (**60**)	NCI 60 cells	GI_50:_ 7 ± 2 nM; 60 ± 25 nM	[56]
	callipeltoside B (**61**), C (**62**)	NSCLC-N6 cells	IC_50_: 15.1, 30.0 μg/mL	[60]
	arenolide (**67**)	HCT-116, A2780 cells	IC_50_: 21, 9.8 mM	[62]
	30-hydroxymycalolide A (**68**), 32-hydroxymycalolide A (**69**), 38-hydroxymycalolide B (**70**)	L1210 cells	IC_50_: 0.019, 0.013, 0.015 μg/mL	[63]
	NA (**76**), NB (**77**), NC (**78**), ND (**79**) and NE (**80**)	P388, P388dox, KB tumor cells	undetermined	[66]
	spongidepsin (**87**)	J774.A1, HEK-392, WEHI-164 cells	IC_50_: 0.56, 0.66, 0.42 μg/mL	[71]
	dactylolide (**88**)	L1210,SK-OV-3 cells	IC_50_: 3.2 μg/mL	[72]
	neohalichondramide (**101**), (19Z)-halichondramide (**102**)	K562 cells	LC_50_: 4.9 μg/mL	[81]
	lasonolides C–E (**106**–**108**)	A-549,PANE-1 cells	IC_50_: 0.13, 4.5, 0.31 μM; 0.38. 4.89, 0.57, 15.6 μM	[83]
	leiodolides A (**112**) and B (**113**)	HCT-116 cells	IC_50_: 1.4, 3.8 μg/mL	[86,87]
	tedanolide C (**114**)	HCT-116 cells	IC_50_: 0.057 μg/mL	[88]
	kabiramide F–I (**115**–**118**)	NCI cells	undetermined	[89]
	swinholide I (**120**), hurghadolide A (**121**)	HCT-116 cells	IC_50_: 5.6, 365 nM	[91]
	oxalatrunculin B (**122**)	HepG2, HCT-116,1301 cells	undetermined	[92]
	neopeltolide (**123**)	A-549, NCI-ADR-RES, P388 cell lines	IC_50_: 1.2, 5.1, 0.56 μg/mL	[93]
	phorbaside C (**134**)	HCT-116 cells	IC_50_: 2 μM	[97]
	tausalarin C (**147**)	K562 cells	IC_50_: 1 μg/mL	[102]
	enigmazole A (**153**)	IC-2	IC_50_: 0.37 μg/mL	[105]
	callyspongiolide (**168**)	Jurkat J16 T, Ramos B lymphocytes	IC_50_: 70, 60 nM	[111]
	phormidolides B (**169**), C (**170**)	A-549, HT-29, MDA-MB-231 cells	undetermined	[112]
	poecillastrins E (**171**), F (**172**), G (**173**)	3Y1 cells	IC_50_: 6.7, 1.2, 5.0 ng/mL	[113]
	macrosphelide M (**180**)	HL-60 cell	IC_50_: 33.2 μM	[120]
	12,13-deoxyroridin E (**191**)	HL-60, L1210 cells	IC_50_: 25, 15 μg/mL	[126]
	myrothecines H, I (**270**–**271**)	HepG-2 cells	IC_50_: 8, 0.4 μM	[156]
	marinomycins A–D (**283**–**286**)	60 cell line panel	LC_50_: 0.005–50 μM	[165]
	arenicolide A (**287**)	KB cells	IC_50_: 30 μg/mL	[166]
	halichoblelide B (**293**)	P388 cell line	ED_50_ 0.63	[169]
	juvenimicin C (**303**)	Hepa 1c1c7 cells	undetermined	[173]
	astolides A (**311**), B (**312**)	K-562, Pgp-positive MDR subline K-562/4	IC_50_: 1.2–1.4 μM	[179]
	biselyngbyolide A (**330**),	HeLa S3, HL60 cells	IC_50_: 0.22, 0.027 μM	[190]
	biselyngbyolide B (**331**)		IC_50_: 3.5, 0.82 μM	[191]
	amphidinolide E (**339**)	L1210, L5178Y cells	undetermined	[194]
	amphidinolide G,H (**341**–**342**)	L1210, KB cells	IC_50_: 0.0054, 0.00048 μg/mL; 0.0059, 0.00052 μg/mL	[197]
	amphidinolides O (**351**), P (**352**)	L1210, KB cells	IC_50_: 1.7, 1.6 μg/mL; IC_50_: 3.6, 5.8 μg/mL.	[208]
	amphidinolide Q (**353**)	L1210 cells	IC_50_: 6.4 μg/mL	[210]
	amphidinolides R (**354**), S (**355**)	L1210, KB cells	IC_50_: 1.4, 4.0 μg/mL; IC_50_: 0.67, 6.5 μg/mL	[213]
	amphidinolide C3 (**357**)	P388, L1210, KB cells	undetermined	[215]
	amphidinolide X (**371**)	L1210, KB cells	IC_50_: 0.6, 7.5 μg/mL	[226]
	amphidinolides B6 (**374**), B7 (**375**)	DG-75 cells	IC_50_: 0.02, 0.4 μg/mL	[227]
	amphidinolide C2 (**376**)	L1210, KB cells	IC_50_: 0.8, 3 μg/mL	[228]
	caribenolide I (**377**)	HCT-116, HCT 116/VM 46,P388	IC_50_: 1.6 nM, 1.6 nM, 0.03 mg/kg	[229]
	iriomoteolide-2a (**387**)	DG-75, cells	IC_50_: 0.006, 0.03 μg/mL	[238]
	iriomoteolide-3a (**388**)	DG-75 cells	IC_50_: 0.08 μg/mL	[239]
	iriomoteolide-4a (**389**), -5a (**390**)	DG-75 cells	IC_50_: 0.8, 1.0 μg/mL	[240]
	iriomoteolide-9a (**391**), -11a (**392**)	HeLa cells	IC_50_: 15, 2 μM	[241]
	iriomoteolide-10a (**393**)	HeLa, DG-75, MH134 cells	IC_50_: 1.5, 1.2, 3.3 μM	[242]
	iriomoteolide-12a (**394**)	DG-75 cells	IC_50_: 50 μM	[242]
	bromophycolide A (**411**)	A2780 cells	IC_50_: 6.7 μM	[258]
	bromophycolide H (**419**)	DU4475 cell line	IC_50_: 3.88 μM	[259]
	bromophycolides J–Q (**421**–**428**)	BT-549, DU4475, MDA-MD-468 et al.	IC_50_: 2.1–7.2 μM	[260]
	bromophycolide K (**425**)	DU4475 cell line	IC_50_: 1.5 μM	[260]
	bryostatin 10 (**458**)	P388 cell line	ED_50_: 0.33 μg/mL	[277]
	bryostatins 16 (**459**), 17 (**460**), 18 (**461**)	P388 cell line	ED_50_: 0.0093, 0.019, 0.033 μg/mL	[282]
	aplyronines D–H (**469**–**473**)	HeLa S_3_ cells	IC_50_: 0.075, 0.18, 0.19, 0.12, 9.8 nM	[286]
	dolabelide A (**474**), dolabelide B (**475**)	HeLa S_3_ cells	IC_50_: 6.3, 1.3 μg/mL	[287]
	dolabelides C (**476**), D (**477**)	HeLa S_3_ cells	IC_50_: 1.9, 1.5 μg/mL	[288,289]
	iejimalides C (**487**) and D (**488**)	KB, L1210 cells	IC_50_: 4.7, 0.2 μg/mL; 10, 0.58 μg/mL	[298]
	lobatamides A–F (**489**–**494**)	NCT’S 60 cells	mean panel GI_50_’s 1.6 nM	[301,302]
	biselides A (**496**), C (**497**)	NCI-H460, MDA-MB-231 cells	IC_50_: 3.53, 3.72 μM; IC_50_: 18.0, 25.5 μM	[303]
	palmerolide A (**501**)	HCC-2998, RXF 393	LC_50_: 18, 6.5, 6.5 μM	[305]
Antibacteria ^a^	curvulone A (**221**)	*B. subtilis*, *Microbotryum, violaceum*, *Septoria tritici*, *Chlorella fusca*	undetermined	[139]
	thiocladospolides F–J (**264**–**268**)	*Edwardsiella tarda*	MIC: 4 μg/mL	[154]
	marinomycins A–D (**283**–**286**)	MRSA, VREF	MIC: 0.1–0.6 μM	[165]
	11′,12′-dehydroelaiophylin (**305**)	MRSA, vancomycin-resistant *Enterococci* pathogens	MIC: 1–4 μg/mL	[175]
	anthracimycin (**308**)	*Bacillus anthracis* (strain UM23C1–1)	MIC: 0.031 μg/mL	[177]
	bromophycolides A (**411**), B (**412**)	MRSA and VREF	MIC: 5.9, 5.9 μM; 5.9, 3.0 μM	[258]
	bromophycolides P–Q (**427**–**428**)	MRSA and VREF	MIC: 1.4, 13 μM; 1.8, 5.8 μM	[260]
Antifugal ^a^	leucascandrolide A (**48**)	*C. albicans*	undetermined	[48]
	neohalichondramide (**101**), (19Z)-halichondramide (**102**)	*C. albicans*	12.5 mm at 25 μg/disk	[81]
	neopeltolide (**123**)	*C. albicans*	MIC: 0.62 μg/mL	[93]
	BK223-A (**181**) BK223-B (**182**), BK223-C (**183**)	*Botrytis cinerea*, *Phoma lingam*, *Phoma bataem*, *Pyrenophora teres*, *Sclerotinia sclerotiorum, Moilinia fructigena, Ascochyta pisi and Alternaria alternata*	undetermined	[121]
	15G256ɩ (**197**),15G256w; (**198**)	*Neuropora crassa* OS-1	undetermined	[128]
	Astolides A (**311**), B (**312**)	*C. albicans, A. niger 219*, *C. tropicales*	MIC: 4, 8 μg/mL	[179]
	bromophycolides A (**411**), B (**412**)	*C. albicans*	MIC: 6.7, 27.7 μM	[258]
	bromophycolides F, I (**417**, **420**)	amphotericin B-resistant *C. albicans*	undetermined	[259]
Antimitotic	halistatin 1, 2 (**15**–**16**)	Inhibition of tubulin polymerization	undetermined	[23,24]
	spirastrellolide A (**94**)	accelerating the entry of cells into mitosis	IC_50_: 100 ng/mL	[79]
Antiviral	bromophycolides A (**411**)	HIV strains 96USHIPS7 and UG/92/029 inhibition	IC_50_: 9.1,9.8 μg/mL	[258]
Antiplasmodial	kabiramide L (**119**)	Against *P. flaciparum* K1	IC_50_: 2.6 μM	[90]
Antiparasite	bromophycolides R–U (**429**–**432**)	Against *Pla. falciparum.*	IC_50_: 0.9–8.4 μM	[261]
VCAM ^b^ inhibition	halichlorine (**47**)	Inhibition to VCAM-1	IC_50_: 7 μg/mL	[47]
Prevent fertilization	exiguolide (**111**)	Inhibited fertilization of sea urchin gametes	IC_50_: 21 μM	[84]
NFκB inhibition	fijiolides A (**309**)	Reducing TNF-α-inducing NFκB activation	IC_50_: 0.57 μM	[178]
Prevent fertilization	oscillariolide (**321**)	Inhibited fertilization of echinoderm eggs	IC_50_: 0.5 μg/mL	[182]
Molluscicidal activity	cyanolide A (**329**)	Against the snail vector *B. glabrata*	LC_50_: 1.2 μM	[189]
Vasoconstrict-ors	zooxanthellatoxins A (**380**), B (**381**)		undetermined	[232,233]
Fast-acting toxin	prorocentrolide B (**382**)	Rapid toxic response in the mouse bioassay	undetermined	[234]
	symbiodinolide (**395**)	Voltage-dependent N-type Ca^2+^ channel-opening activity	IC_50_: 7 nM	[14]
	acuminolide A (**396**)		IC_50_: 10^−6^ M	[246]
Prevent fertilizatoin	haterumalide B (**495**)	Inhibited fertilization of sea urchin eggs	IC_50_: 0.01 μg/mL	[302]

^a^ In the pharmacology column, cytotoxic, antibacteria and antifungal parts present species to which the compounds show inhibition bioactivities. ^b^ Vascular cell adhesion molecule.

## 3. Conclusions and Outlook

This review presents a summary of 505 marine-derived macrolides reported from 1990 to 2020 and highlights their chemical and biological diversity. As shown in Figure 1, sponges are the dominant producer of marine macrolides, yielding 173 of these 505 compounds (34.3%). Fungi and dinoflagellates are also important sources, producing 19.4% and 12.1%, respectively, of the macrolides reviewed. Marine animals (cnidarians, bryozoans, tunicates, and mollusks) produced significantly fewer macrolides with a combined percentage of 11.6%, while marine plants (red algae) yielded 9.5%. Marine microbes (including fungi, bacteria, cyanobacteria) produced 32.7% of 505 macrolides. Notably, macrolides obtained from sponges have fallen since 2010, while microbes, especially fungi, have grown to be important producers (Figure 2). This phenomenon suggests that biochemists are acknowledging that sampling slow-growing sessile organisms to identify natural products is not an eco-friendly practice. More attention is now being given to microbes due to their capacity for unlimited reproduction and the ease with which their genome can be mined for targeted metabolites. Marine macrolides have a broad range of properties, including cytotoxic, antifungal, antimitotic, and some other activities (Table 1). Cytotoxicity is their most significant bioactivity, highlighting that marine macrolides include many potential antitumor drug leads. 

For macrolides with larger macrocyclic rings, such as reidispongiolides A and B [34], symbiodinolide [14] and zooxanthellatoxins A and B [232,233], the flexible ring structures make stereochemistry identification more difficult. Novel configuration determination technologies, such as sponge crystals [309], are needed to solve this problem. Although they possess diverse bioactivities, few marine macrolides have been developed into approved antitumor drugs or even for clinical trials during the last thirty years. Limited production from natural biomaterials and difficulties in synthesis may be hindering new drug discovery. High throughput screening and investigation of target prediction and additional bioactivity mechanisms must be employed to increase the successful discovery of lead compounds from marine macrolides. This should include mining for more structurally unusual macrolides with broader bioactivities.
